# “Bottled” spiro-doubly aromatic trinuclear [Pd_2_Ru]^+^ complexes†

**DOI:** 10.1039/d0sc04469e

**Published:** 2020-10-23

**Authors:** Maksim Kulichenko, Nikita Fedik, Anna Monfredini, Alvaro Muñoz-Castro, Davide Balestri, Alexander I. Boldyrev, Giovanni Maestri

**Affiliations:** Department of Chemistry and Biochemistry, Utah State University Logan UT 84322 USA a.i.boldyrev@usu.edu; Department of Chemistry, Life Sciences and Environmental Sustainability, Università di Parma Parco Area delle Scienze 17/A 43124 Parma Italy; Grupo de Química Inorgánica y Materiales Moleculares, Facultad de Ingeniería, Universidad Autonoma de Chile El Llano Subercaseaux 2801 Santiago Chile

## Abstract

Following an ongoing interest in the study of transition metal complexes with exotic bonding networks, we report herein the synthesis of a family of heterobimetallic triangular clusters involving Ru and Pd atoms. These are the first examples of trinuclear complexes combining these nuclei. Structural and bonding analyses revealed both analogies and unexpected differences for these [Pd_2_Ru]^+^ complexes compared to their parent [Pd_3_]^+^ peers. Noticeably, participation of the Ru atom in the π-aromaticity of the coordinated benzene ring makes the synthesized compound the second reported example of ‘bottled’ double aromaticity. This can also be referred to as spiroaromaticity due to the participation of Ru in two aromatic systems at a time. Moreover, the [Pd_2_Ru]^+^ kernel exhibits unprecedented orbital overlap of Ru d_*z*^2^_ AO and two Pd d_*xy*_ or d_*x*^2^−*y*^2^_ AOs. The present findings reveal the possibility of synthesizing stable clusters with delocalized metal–metal bonding from the combination of non-adjacent elements of the periodic table which has not been reported previously.

## Abstract 2

Synthesis of a triangular [Pd_2_Ru]^+^ complex with delocalized metal–metal bonding between non-adjacent elements of the periodic table, double aromaticity and overlap of d-AOs with different angular momentum.

## Graphical abstract

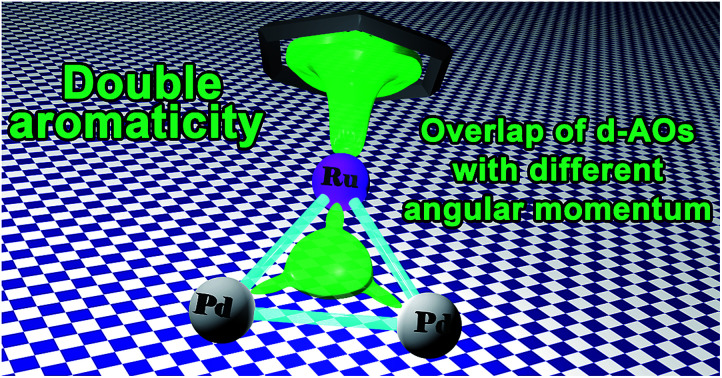

## Introduction

For more than a hundred years of its existence, aromaticity has proven itself to be an extremely useful and reliable concept^1–6^ which enables the explanation of bonding patterns in many non-trivial chemical systems. Being widely utilized, the concept was significantly extended beyond classic organic compounds since the introduction of Hückel's 4*n* + 2 rule in 1931.^7,8^ Interestingly, the expansion of aromaticity to a wide range of inorganic and all-metal systems led to heated discussions^9–11^ in the scientific community. In 2018 Saito *et. al.* made a game changing discovery which put an end to all doubts regarding aromaticity and its transferability throughout different branches of materials chemistry. They managed to synthesize a doubly aromatic compound [C_6_(SePh)_6_]^2+^ that possesses cyclic σ-symmetric and π-symmetric aromatic rings.^5^ This milestone indicates that other types of aromaticity apart from the conventional π-type are not elusive. Obviously, even a hundred years later, the paradigm of aromaticity is undergoing alterations and expansions. All-metal aromaticity^12–19^ is a well-known concept that enables describing the bonding properties of all-metal compounds similar to those involving main-group elements.^20,21^

**Fig. 1:**
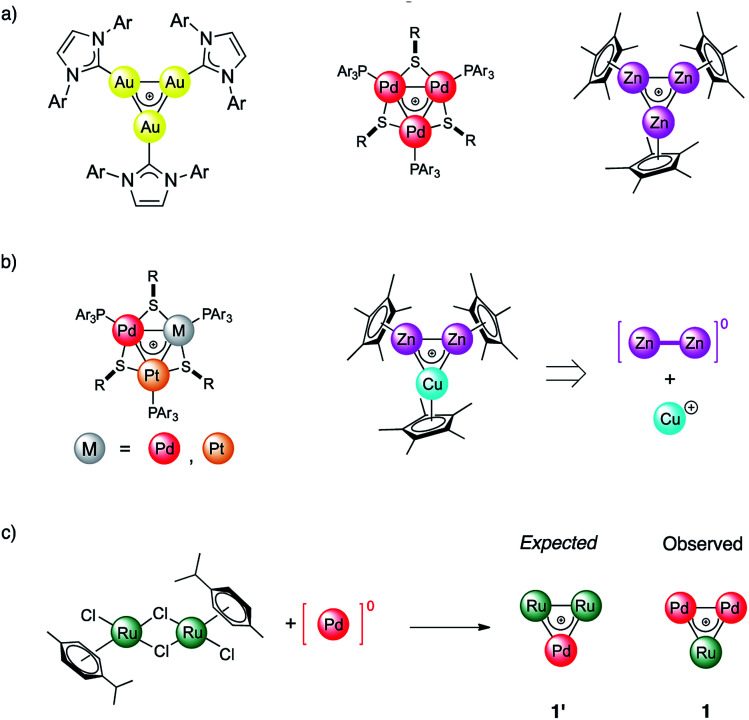
(a) Homonuclear triangular complexes with delocalized bonding involving d-block transition metals; (b) reported examples of corresponding heterobimetallic species involving adjacent elements of the periodic table; (c) present work: combination of Ru and Pd in a cluster with delocalized metal–metal bonding.

As a matter of fact, the majority of metal aromatic structures can exist only in laser beams and only for a very short time. Among transition metal (TM) complexes, TM triangles were recently found to be aromaticity carriers that can mimic aromatic donor ligands.^22–26^ All-metal aromaticity in stable triatomic hetero-metallacycles was reported for Ga_3_,^27^ Au_3_,^28^ Zn_3_,^29^ Pd_3_,^22^ and Pt_3_ complexes as well as in Zn_2_Cu and Pd–Pt mixtures (Fig. 1).^23^ Being caught in the bulk as stable liganded cations, they are not limited by the short lifetime which, opens a wide range for the exploitation of their unique stereoelectronic properties in catalysis and materials chemistry.^26,30^ We recently showed that triangular metal complexes exhibit a spectacular ability to convert unsaturated linear organics into tricyclic complexes. This is achieved through selective C–C activation which prior to our discovery was inaccessible by other discrete Pd catalysts.^31^ Remarkably a trinuclear framework of noble metals can also effectively catalyze alkynes to *cis*-alkenes through a “green” route without additional organic solvents.^26^ Moreover, trinuclear all-metal cores could be easily tailored by organic ligands to deliver diversity and stability. Beyond clusters, σ-aromaticity could be a driving force for stabilization in periodic systems. For instance, MoS_2_ was recently shown to possess TM-based triangular σ-aromatic bonds delocalized over three Mo atoms inside every hexagonal ring.^32^

In this work we report the synthesis and bonding analysis of hetero-metallacycle [RuPd_2_]^+^ complexes. These clusters are bench-stable in the solid state and their metal cores display a doubly aromatic character (Fig. 1c) with an encapsulated TM triangle. This triangle is a direct descendant of a recently discovered family of pure and mixed Pd and Pt triangular cations (Fig. 1a and b),^22,23,25,26^ whose noble-metal core is an analogue of the cyclopropenyl cation. It is worth noting that reported examples of heterobimetallic triangles featuring delocalized metal–metal bonding are limited at present to combinations of neighboring atoms of the periodic table.^29,35^ Our findings thus show that this limit could be overcome. This opens a new avenue for the potential combination of a variety of transition metal nuclei into stable triangular metal-aromatic frameworks.

The central unit of the newly obtained structure is a Ru atom which is the vertex of the [RuPd_2_]^+^ triangle being the first example of mixed triangles among these metals. Moreover, Ru is involved in both σ- and π-aromaticity. σ-aromaticity is realized as the d-AO based multicenter bond which is delocalized over the inner [RuPd_2_]^+^ triangle with considerable contribution from the sulfur electron density. Besides, transition metals are known to interact with the electron cloud of benzene which, in turn, leads to the charge transfer and formation of conjugated electron density between the TM and benzene. The second type of aromaticity, π-aromaticity, is caused by this interaction and represented *via* d-AOs of Ru interacting with the benzene electron cloud. The doubly aromatic behavior is supported by MO and AdNDP (Adaptive Natural Density Partitioning) analyses. This doubly aromatic bonding pattern can also be described in terms of spiroaromaticity^33,34^ where, according to the original notation, aromatic rings are fused by sharing a single atom rather than by a mutual bond. Indeed, as we will see later, Ru is a full participant in the benzene π-cloud while not being a structural part of the six-membered carbon ring.

Doubly aromatic species have been a topic of perpetual study both in computational design^35–41^ and photoelectron spectroscopy experiments yielding novel but “non-bottled” short lived clusters.^42–45^ However, to the best of our knowledge, none were obtained as stable phases in the bulk except for Saito's.^5^ Thus, the liganded triangular TM-based complex reported here is the second “bottled” example of double aromaticity which is another milestone towards expansion of the aromaticity concept throughout the chemistry space. Moreover, this is the first reported system with the Ru atom taking part in two types of aromaticity simultaneously.

## Experimental methods

Pd(dba)_2_, ruthenium dimers, phosphines and silver salts were purchased from commercial sources and used as received. Sodium thiolates were obtained by reducing the corresponding thiols with sodium hydride. Solvents were degassed by bubbling N_2_ for at least 30 minutes prior to use. Reactions and filtrations were carried out under N_2_ using the standard Schlenk technique.

## Computational methods

The computational characterization of the experimental structure and its truncated model was performed using the Gaussian16 software package.^46^ The experimental structure was optimized using the PBE0-GD3BJ^47,48^ hybrid functional accounting for Grimme's dispersion correction. This method was shown to produce reliable energetics (compared to CCSD(T)/CBS or large basis sets) and geometries for a wide range of organometallic systems containing heavy elements.^49^ We employed the mixed basis set 6-31+G*^50^ for all “organic” elements (H, C, S, and P) and Def2-TZVPP^51^ with ECP for heavy elements (Pd and Ru). Optimized structures were revealed to be energy minima without any imaginary frequencies. The optimized geometry did not differ substantially from the experimental one, especially in the triangular core. The differences between experimentally resolved bond lengths and calculated ones were within 0.03 Å. The same holds true for the truncated model. We assume that this indicates a proper selection of the level of theory. Some slight discrepancies between experimental and calculated structures are related to the gas-phase approximation (*i.e.*, absence of crystal packing effects) and reduction of stereochemical “pressure” during optimization. Overall, the optimized structure preserves the shape and bonds in the all-metal core, while conformational rotations of organic substituents are not important. To be completely unbiased we conducted a bonding study on top of the experimental and theoretical geometries and, as we expected, it yielded the same results. It is widely known that transition metals and related complexes tend to exhibit multireference character; however, all structures computed in the article are closed-shell singlets, according to internal wave function stability tests (stable = opt). Additionally, CASSCF(6,6)^52,53^ calculations were performed for the truncated model system in the ORCA4.2 package,^54,55^ and the coefficient of the Hartree–Fock solution was found to be 0.910 while coefficient at second configuration was only 0.031. In addition, the HOMO occupancy in the model system is equal to 1.89|*e*|. Thus, we can say without a shadow of a doubt that the structure studied (and its model system) are closed-shell singlets. Chemical bonding analysis was performed using the AdNDP^56^ (Adaptive Natural Density Partitioning) code which is an extension of the NBO^57–59^ and enables assignment of multicenter *n*c–2e bonds (*n* > 2). AdNDP was successfully applied for bonding deciphering in a variety of chemical systems including solids and even solvated ions.^60–67^ We previously showed that AdNDP is a tool almost independent of method/basis set combinations.^12,68^ Recently, we also found that relativistic effects have a negligible^12^ effect on bonding pictures, thus significantly simplifying calculations for transition metals and their complexes. Several functional/basis–set combinations were tested for the bonding picture to be unbiased: PBE0/Def2-TZVP,^47^ MN15-L/Def2-TZVP, PBE0/Sapporo-TZP, MN15-L/Sapporo-TZP,^69,70^ M06-2X/Def2-TZVP,^71^ and PBE0/cc-pVDZ.^72^ All of them gave essentially the same results.

The induced magnetic field (*B*^ind^) upon application of an external magnetic field (*B*^ext^)^73,74^ was obtained as a grid of the nucleus independent shielding tensor (*σ*_ij_), where *B*_i_i^nd^ = −*σ*_ij_*B*^ext^_j_, resulting in shielding behavior at the center of the ring as useful magnetic criteria of aromaticity.^75–78^ For convenience, the i and j suffixes are related to the *x*-, *y*- and *z*-axes of the molecule-fixed Cartesian system (*i*, *j* = *x*, *y*, *z*). The values of *B*^ind^ are given in ppm in relation to *B*^ext^ calculated within the GIAO formalism as implemented in the ADF2019 code^79^ employing the OPBE functional^80^ and frozen core STO-TZ2P basis set, leaving valence electrons to be treated variationally.

## Results and discussion

**Fig. 2:**
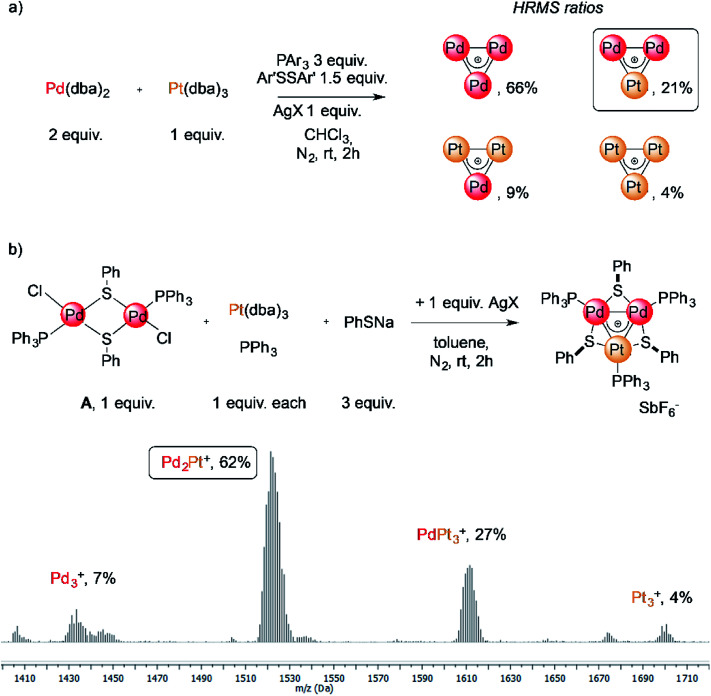
(a) The unselective reaction of Pd(0) and Pt(0) precursors for the synthesis of heterobimetallic triangles; (b) the stepwise assembly from a dimer and a monomer that triggered the present unexpected findings.

The reaction of low-valent palladium and platinum precursors allows synthesis of the corresponding heterobimetallic triangles in the presence of a tertiary phosphine, a disulfide and a silver salt. However, the process is not selective, leading to the formation of four triangular complexes (Fig. 2a).^23^ This can be observed by analyzing the reaction mixture using MS analysis through the isotopic fingerprint of two homonuclear Pd_3_^+^ and Pt_3_^+^ complexes and of two heterobimetallic Pd_2_Pt^+^ and PdPt_2_^+^ species (see the ESI†). The isolation of pure species requires a rather lengthy sequence of multiple chromatographic separations on silica gel, limiting in turn the synthetic viability of the method. According to literature studies, the assembly of an M_3_^+^ complex could be in principle achieved through the reaction of a suitable dimer with a mononuclear precursor.^28–30,81,82^ We tried to test this approach using an oxidized dimer and a low-valent monomer for selective preparation of heterobimetallic complexes. We thus mixed a model Pd(ii) dimer with bridging thiolates (A)^83^ with the platinum(0) precursor Pt(dba)_3_ (dba = dibenzylideneacetone) under our model conditions. Gratifyingly, the desired heterobimetallic complex Pd_2_Pt^+^ species became the most abundant product (Fig. 2b), confirming the working hypothesis.

Focussing on this finding, we then thought to exploit this strategy to assemble novel heterobimetallic triangular complexes. Among these species, reported examples featuring metal aromaticity are limited at present to neighboring elements in the periodic table, such as palladium and platinum for group VIII or copper and zinc among first-row elements.^23,29^ We thus believed that the synthesis of a heterobimetallic trinuclear complex incorporating two different, nonadjacent transition metals would be a challenging and prominent goal. In a preliminary experiment, Ru(ii) dimer B was mixed with an equimolar amount of Pd(dba)_2_, diphenyldisulfide and triphenylphosphine in chloroform (0.01 M). Analysis of the crude mixture by MS revealed that no traces of any putative Ru_2_Pd species formed (1′). Much to our surprise, however, the diagnostic isotopic pattern of an unexpected [RuPd_2_]^+^ complex appeared instead (1). To the best of our knowledge, no example of mixed heterobimetallic triangles of ruthenium and palladium have been described yet.

**Fig. 3:**
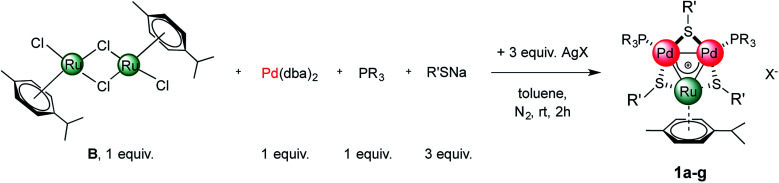
Synthesis of heterobimetallic [RuPd_2_]^+^ complexes.

We therefore tried our best to characterize this unexpected product and to test the generality of the reaction. Upon a lengthy series of frustrating experiments, we found that the best conditions were achieved by mixing Pd(dba)_2_ with 2 equiv. of Ruthenium(ii) dimer B, 3 equiv. of sodium thiolate, 1 equiv. of a tertiary phosphine and 3 equiv. of a silver(i) salt in toluene (Fig. 3, Table 1, see the ESI† for details). The reagents were mixed under a nitrogen atmosphere at room temperature for two hours. Upon a routine work-up, purification of the resulting mixture by column chromatography on silica gel followed by crystallization of the fraction rich in the desired complex by means of vapor diffusion led to the formation of dark green crystals of [RuPd_2_]^+^ complex 1. Upon purification, these species became bench-stable in the solid state. In contrast, they tended to decompose in solution within a few days. Try as we might, we were unable to achieve higher yields of the pure heterobimetallic complexes (see the ESI† for details), although the synthetic method proved rather general varying either the thiolate, the phosphine or the silver salt (Fig. 3 and Table 1). The main byproducts of the reaction are the homonuclear Pd_3_^+^ complex and a Ruthenium dimer in which thiolates replaced the chlorides of B.^84^ The latter has a very similar polarity compared to the heterobimetallic cluster, thus hampering straightforward purification of the desired complex. This is confirmed by the low isolated yield of pure complex 1a (13%, entry 1), for which the purification was particularly hindered. The replacement of *p*-tolyl with a phenyl group on the thiolate proved beneficial to this end, allowing us to isolate 1b in 32% yield (entry 2). The replacement of SbF_6_^−^ with PF_6_^−^ as the counterion was tolerated by the process (1c, entry 3). The lowest yield (6%) was obtained with *p*-tolyl groups both on the phosphine and on the thiolate, suggesting that the presence of electron-donating groups on all ligands could hamper the formation of the cationic complex (1d, entry 4). Thiolates with different halides at their para position were also tested, giving comparable results (entries 5–7, 14–36% for 1e–g). The present method is limited to aromatic substituents on phosphines and thiolates, attempts with aliphatic ones being unsuccessful.

**Table 1:** Synthesis of heterobimetallic [RuPd_2_]^+^ complexes^a^

Entry	R	R′	X	Yield (%)
1	Ph	Ph	SbF_6_	13 (26^b^)
2	4-Me-C_6_H_4_	Ph	SbF_6_	32
3	4-F-C_6_H_4_	Ph	PF_6_	16
4	4-Me-C_6_H_4_	4-Me-C_6_H_4_	SbF_6_	6
5	4-Me-C_6_H_4_	4-Br-C_6_H_4_	SbF_6_	20
6	4-F-C_6_H_4_	4-Cl-C_6_H_4_	SbF_6_	14
7	4-Me-C_6_H_4_	4-Cl-C_6_H_4_	SbF_6_	36

a Reaction conditions: on a 0.16 mmol scale, 0.01 M in toluene, under N_2_ at room temperature for two hours; isolated yields with column chromatography on silica gel and crystallization by vapor diffusion.

b ^1^H NMR yield.

The behavior in solution of [RuPd_2_]^+^ complexes has both analogies and differences compared to their homonuclear peer [Pd_3_]^+^. In contrast to *C*_3_-symmetric M_3_^+^ complexes for which NMR analyses showed a single set of resonances corresponding to the asymmetric unit of the cluster, those for the present [RuPd_2_]^+^ ones show different sets of signals. Phosphines generate two coupled doublets in the ^31^P NMR spectrum, as well as two patterns of signals in the ^1^H NMR spectrum, for the resonances of their Ar–H fragments. Thiolates follow suit. In particular, those between palladium and ruthenium nuclei give resonances that are shifted downfield compared to that of the thiolate between the two palladium atoms. The UV-vis absorption spectra of Pd_2_Ru^+^ complex 1a is similar to that of the M_3_^+^ ones in the region up to 400 nm, in which both display two intense bands (*ε* around 10^4^ M^−1^cm^−1^). However, an additional difference emerges at higher wavelength, as the present heterobimetallic species display a new, weaker band in the visible region at around 600 nm (*ε* = 3.8 × 10^3^ M^−1^cm^−1^, spectra in the ESI†).

**Fig. 4:**
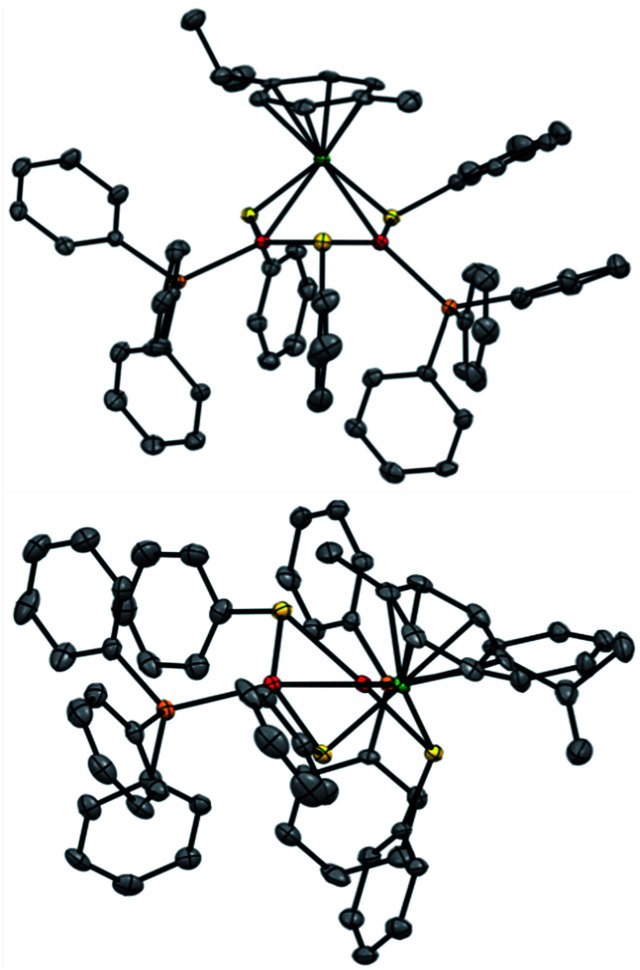
The crystal structure of Pd_2_Ru complex 1a; ellipsoid drawn at 50% probability, hydrogen atoms and SbF_6_^−^ anion omitted for clarity; top view (up) and side view (down); Pd (red), Ru (green), S (yellow), P (orange), and C (gray).

Regarding solid-state analysis, crystals of 1a were then analyzed using X-rays (Fig. 4). The complex has a metal core composed of two Pd atoms and one Ru atom, which are coordinated to two phosphines and a *p*-cymene, respectively. Three thiolates act as bridging ligands between the metal atoms. The cluster has an overall positive charge that is balanced by a non-coordinating counteranion that lies far away from the metal core. In contrast to the Pd_3_^+^, Pt_3_^+^ and Pd_*x*_Pt_3−*x*_ complexes, [RuPd_2_]^+^ complex 1a has no *C*_3_ symmetry anymore. Indeed, the Pd–Ru distances are 2.7430(6) and 2.7692(7) Å, respectively, and the Pd–Pd one is 2.7379(7) Å. The three angles of the triangular core are 59.56(2)°, 59.74(2)° and 60.69(2)°. These results show that the central metallic core of the [RuPd_2_]^+^ complex is no longer a perfect equilateral triangle but slightly deviates from it. The Ru–S–Pd and Pd–S–Pd angles are 73.52(5)°, 73.68(5)° and 72.45(5)°, respectively. The arrangement of ligands is in sharp contrast with that observed in Pd_3_^+^ complexes, where all the heteroatoms, which complete the first coordination sphere of palladium nuclei, are essentially coplanar with respect to the metal core. Regarding the [RuPd_2_]^+^ complex, the three thiolates point above and below the plane of the triangle, with dihedral angles of 95.27(4)°, 97.33(4)° and 96.88(4)°, respectively. The six metal–sulphur distances show little difference among them, ranging between 2.28 and 2.32 Å. These values are comparable with those observed in related homonuclear triangular clusters. Phosphines are slightly less tilted compared to the metal core, with dihedral angles of 150.15(5)° and 157.66(5)°. The palladium-phosphorous distances are nearly identical (2.279(5) and 2.282(4) Å, respectively) and display values similar to those of Pd_3_^+^ complexes. The C–C distances between C(sp^2^) atoms of the cymene unit range within a narrow interval (between 1.39 and 1.42 Å). Ruthenium–carbon ones follow suit (2.20–2.25 Å). These results suggest that the aromatic carbocycle coordinates with the metal in an *η*^6^ fashion.

The most striking structural difference compared to previous examples of homonuclear M_3_^+^ complexes (M = Pd, Pt) lies in the metal–metal distances of present [RuPd_2_]^+^ clusters. Although the atomic radius of Ru is just 0.03 Å smaller than that of palladium, the Pd–Pd and Pd–Ru distances in 1a are between 0.15 and 0.2 Å shorter than those observed in the structures of their analogous Pd_3_^+^ peers that share otherwise identical bridging and ancillary ligands. We speculated that a different metal–metal bonding interaction could have been present in this case to account for this meaningful structural difference, reasoning that steric demands would fall short in providing any apparent rationale. In particular, we wondered whether the present heterobimetallic complex could be described as either a Pd(i) dimer flanked by a Ru(ii) fragment or a system in which the three metals share the formal +4/3 oxidation state because of a delocalized metal–metal bonding network. Bonding analyses were therefore carried out to gain insights into the factors that triggered the shrinkage of the core of this heterobimetallic triangle compared to its Pd_3_^+^, Pt_3_^+^ and Pd_*x*_Pt_3−*x*_^+^ peers.

Structural optimization did not change the geometry significantly with a deviation in bond lengths of about 0.03 Å. Unlike its Pd_3_^+^ ancestor, the Pd_2_Ru^+^ triangle is not equilateral but rather quasi-isosceles. Both calculated Pd–Ru triangle edges are almost identical: 2.788 Å and 2.813 Å are the experimental ones (2.764 Å and 2.737 Å). The calculated Pd–Pd base is 2.734 Å while the experimental distance is 2.732 Å.

**Fig. 5:**
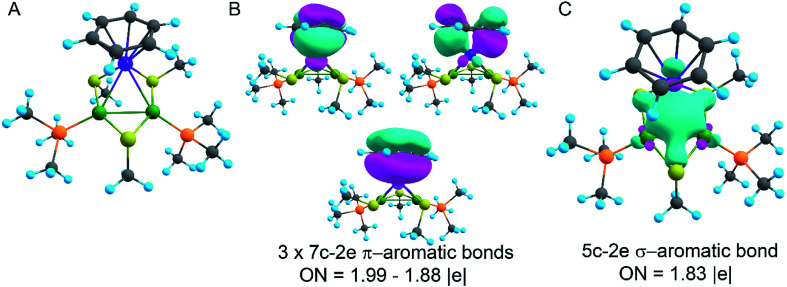
(A) Truncated model of the synthesized compound. Ru-purple, Pd-green, S-yellow, P-orange, C-grey, and H-blue; (B) π-aromaticity pattern recovered by the AdNDP method; (C) σ-aromaticity pattern recovered by the AdNDP method. Classical bonding in organic ligands is omitted for simplicity.

In order to decipher the bonding picture of the liganded triatomic hetero-metallacycle [RuPd_2_]^+^, we utilized the AdNDP localization scheme. The starting point is a simplified truncated model, *i.e.*, all benzene rings are replaced by methyl groups except the one coordinated with the Ru atom (Fig. 5A). As will be shown below, this is a reasonable approximation since all the interesting bonding features are located around the Pd_2_Ru core preserved in the model.

The model of 1a possesses 148 valence electrons meaning the presence of 74 bonding elements. AdNDP recovered 14 lone pairs: four on each Pd with occupation numbers (ONs) 1.97|*e*|, one on each S with ONs = 1.92|*e*|, and three on Ru with ONs = 1.74–1.96|*e*|. Then 56 two-center-two-electron (2c–2e) bonds with ONs = 1.85–1.92|*e*| were found between every two neighboring atoms except Pd–Pd, Pd–Ru, and Ru–C. This gives us 70 bonding elements in total. Therefore, 4 bonds still remain unlocalized. Definitely, three of them should involve the benzene ring (obviously having 6 valence electrons) which, in turn, involves interaction with the directly linked Ru atom. The degree of this interaction can be estimated in terms of occupation numbers. As can be seen in Fig. 5, aromatic pi-bonds are not just highly polarized towards Ru but even have a common electron density with this atom. The localization of three electron pairs over the benzene ring as 6c–2e (without Ru) multicenter bonds results in polarization towards Ru bonds with ONs of 1.52, 1.54, and 1.86|*e*|. The first two numbers – 1.52 and 1.54 – are basically the lowest bar for acceptable occupation numbers and we usually do not trust bonds with such low ONs. Based on our previous work,^12,85^ such a low occupancy most likely indicates that the bond should be delocalized over a greater number of centers. This is the case here because inclusion of Ru, *i.e.*, consideration of 7c–2e bonds accounting for C_6_H_6_–Ru interaction, yields substantially greater values of 1.88, 1.94, and 1.99 |e| (Fig. 5 B). The contribution of Ru to these bonds accounts for 20%, 21% and 7% of electron density, respectively. Besides, the latter ON of 1.99|*e*| is a perfect localization of 2 electrons which is basically MO “occupation” (in single reference closed-shell calculations). These observations give rise to TM-coordinated π-aromaticity inside the system.

One electron pair remains unlocalized to this point. Interestingly, the overall symmetry of the complex is *C*_1_ because of the branched organic ligands. They also tend to slightly distort the internal Pd_2_S_2_Ru kernel. However, if we neglect this distortion, the Pd_2_S_2_Ru core has at least one plane of symmetry, so it could be approximately considered as belonging to the *C*_s_ group. Therefore, the overall complex is quasi-symmetric and it has a somewhat symmetric core. The only bonding combination that satisfies the ON threshold and does not break the quasi-symmetry of the compound is the inner triangle-based 3c–2e σ-bond. Therefore, it complies with the classical 4*n* + 2 rule which was extended from π-aromatics to σ-aromatics.^5,86,87^ According to this rule, aromatic compounds possess 4*n* + 2 electrons participating in aromaticity where *n* is an integer. That is, in our case 4*n* + 2 = 2 where *n* = 0. As we can see in Fig. 5C, the σ-aromatic bond consists of d-AOs of transition metals. In fact, the electron density of this bond is somehow attracted by sulfur since the inclusion of neighboring S atoms increases the ON from 1.30 to 1.83|*e*|.

**Fig. 6:**
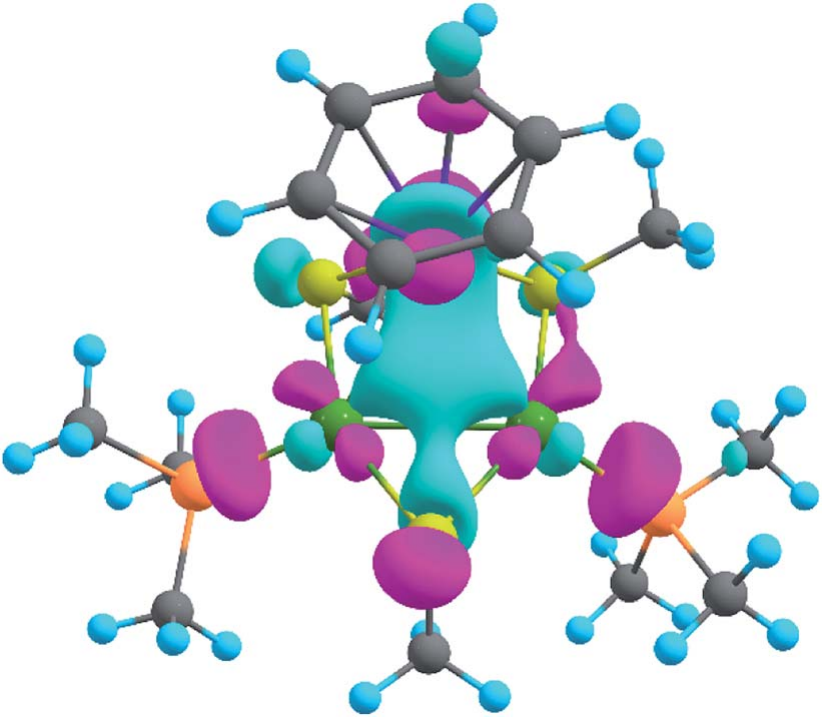
HOMO of the synthesized system. The isovalue is 0.047.

The presence of σ-aromaticity is further supported by the shape of the HOMO (Fig. 6). Its quasi-symmetric form is mostly a 3c–2e bond occupied by the abovementioned 4*n* + 2 = 2 electrons and arises from d-AOs of TMs and corresponds to σ-aromaticity. As we can see, Ru is a common atom of two aromatic systems. This makes the term “spiroaromaticity” valid because in spiro compounds at least two aromatic rings have one (and only one) common atom. However, the Ru atom is not technically a part of the carbon ring plane and is rather π-stacked with the benzene fragment. Therefore, we feel that some variations in the terminology may exist for such unique cases where a few aromatic patterns coexist. Due to the lack of reports on such systems we will leave it to the reader to define the applicability of this term (“spiroaromaticity) based on a geometry or electron density point of view.

Note the very unusual topology of the HOMO: it is constructed by mixing different types of d-AOs. Even visual assessment gives us a hint that the Ru d-AO is d_*z*^2^_ (d_0_) while Pd d-AOs have different angular momentum. A closer look at the AO coefficients reveals that the greater contribution of Pd atoms comes from d_−2_ AOs while for the Ru atom it is indeed d_0_ AO. To the best of our knowledge this might be the first case of such peculiar orbital overlap. Metal-aromatic triangular complexes indeed feature the prevalent combination of a single type of d-AO to form their delocalized MOs.^28,29,33–35^ Analysis of a full model, in which all substituents are present, results in essentially the same occupation numbers and bond shapes.

**Fig. 7:**
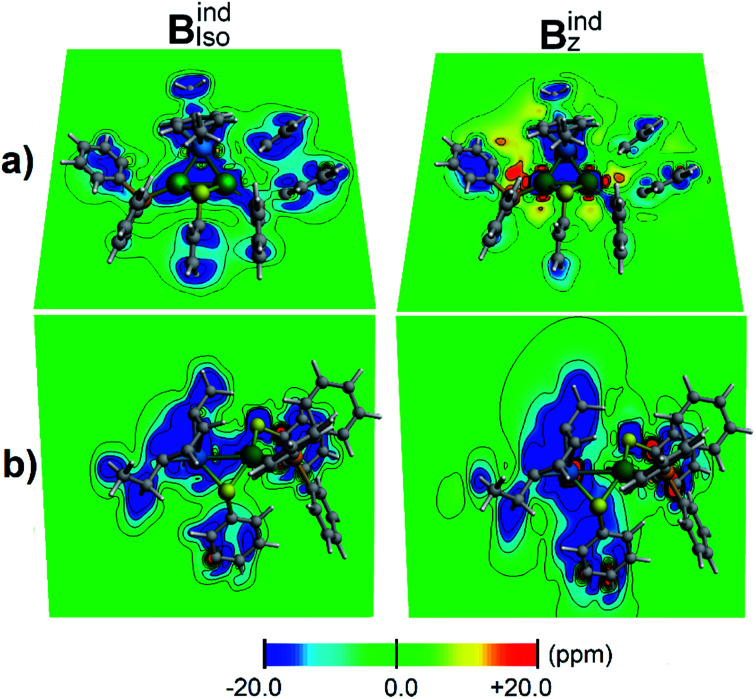
Induced magnetic field of 1a denoting *B*^ind^_iso_ and *B*^ind^_*z*_ oriented perpendicularly to the [Pd_2_Ru]^+^ plane. Two views are given: (a) contour plot contained in the [Pd_2_Ru]^+^ plane and (b) bisecting the Pd–Pd bond. Blue, shielding; red, deshielding. Ru is blue and Pd is green.

According to the magnetic criteria of aromaticity, aromatic species build up a shielding response under an applied external magnetic field. Isotropic (*B*^ind^_iso_) and *z*-axis oriented (*B*^ind^_*z*_) terms are given to provide a picture of the induced magnetic field originating from 3c–2e σ-aromatic and 7c–2e π-aromatic kernels.^75–78^ The isotropic (*B*^ind^_iso_) term, which is an average of the different orientations of the applied field owing to the constant molecular tumbling in solution, exhibits shielding regions ascribed to the heterobimetallic triangular [Pd_2_Ru]^+^ cluster owing to the presence of the 3c–2e σ-aromatic bonding pattern (Fig. 7a). In addition, the 3 × 7c–2e π-aromatic kernel involving the cymene unit and Ru also gives rise to a shielding region (Fig. 7b), supporting the incorporation of the coordinated Ru atom in the π-aromatic system. The shielding regions ascribed to the different aromatic phenyl rings from PPh_3_ ligands are expectedly observed. Upon application of a magnetic field oriented perpendicularly to the [Pd_2_Ru]^+^ ring (*B*^ind^_*z*_), the ring current effect is enabled^78^ depicted as a long-range shielding cone (Fig. 7b) with a complementary deshielding region at the ring contour (Fig. 7a). This agrees with the magnetic criteria of aromaticity providing one more piece of evidence of aromaticity in the [Pd_2_Ru]^+^ kernel. Moreover, the Pd–S–Pd section shows a diminished response from the *B*^ind^_*z*_ term (Fig. 7b), suggesting that the thiolate ligand is not involved in the aromaticity of the [Pd_2_Ru]^+^ ring.

Thus, the 3c–2e multicenter bond in the [Pd_2_Ru]^+^ kernel gives rise to the second type of aromaticity in the structure – all-metal TM-based σ-aromaticity. This makes [Pd_2_Ru]^+^ the first observed example of mixed trinuclear aromatics involving these noble metals. Therefore, the examined compound is just the second example of double aromatics which exist as a stable solid rather than elusive species in a laser beam. Noticeably, no Ru-interfaced double aromatics were previously reported even as short lifetime clusters.

## Conclusions

We established a synthetic route opening new perspectives on obtaining heteronuclear all-metal cores comprising non-adjacent transition metals from the periodic table. The obtained and characterized [Pd_2_Ru]^+^ compound is the first example of a heterobimetallic triangular complex among these noble metals. Transition metal d-AOs with different angular momentum form a conjugated σ-aromatic system delocalized over the inner noble metal triangle supported by AdNDP and magnetic criteria of aromaticity. In addition, the Ru-coordinated benzene ring gives rise to the second type of aromaticity within one compound. Interaction of d-AOs of Ru with benzene π density causes the appearance of 7c–2e π-aromatic bonds highly polarized towards the Ru atom and having common electron density with it. This makes the observed structure the second example of “bottled” double aromaticity which is accessible as a stable solid in contrast to the short lifetime aromatic molecules detected only in laser beams. Moreover, this cluster is the first reported case of Ru involved in two types of aromaticity simultaneously making the term spiroaromaticity also applicable.

## Conflicts of interest

There are no conflicts to declare.

## Supplementary Material

SC-012-D0SC04469E-s001

SC-012-D0SC04469E-s002
