# Supplementary material for: “Bottled” spiro-doubly aromatic trinuclear [Pd2Ru]+ complexes
Source: Chem Sci. 2020 Oct 23;12(1):477–86. doi: 10.1039/d0sc04469e (PMC8178750; doi:10.1039/d0sc04469e)
Supplement: SC-012-D0SC04469E-s001 [file SC-012-D0SC04469E-s001.pdf]

Electronic Supplementary Material (ESI) for Chemical Science.
This journal is © The Royal Society of Chemistry 2020

Electronic Supplementary Information

“Bottled” spiro-doubly aromatic trinuclear $[Pd_2Ru]^+$ complexes

Maksim Kulichenko[a], Nikita Fedik[a], Anna Monfredini[b], Davide Balestri[b], A. I. Boldyrev[a]*, Giovanni Maestri[b]

[a] Department of Chemistry and Biochemistry, Utah State University, Logan, UT 84322, USA

[b] Department of Chemistry, Life Sciences and Environmental Sustainability, Università di Parma, Parco Area delle Scienze 17/A, 43124 Parma, Italy

*a.i.boldyrev@usu.edu

Contents

		Page
1.	General remarks	3
2.	Experimental procedures	4
3.	Extended screening of reaction conditions	5
4.	MS analyses for heterobimetallic complexes	6
5.	Spectroscopic data	8
6.	Summary of crystallographic data	15
7.	Copies of NMR spectra	20
8.	Cartesian coordinates of computed structures	35
9.	Two virtual σ-aromatic MOs	42
10.	References	43

1. General remarks

$Pd(dba)_2$, ruthenium dimers, phosphines and Ag salts were purchased from commercial sources and used as received. Sodium thiolates were obtained reducing the corresponding thiols with sodium hydride. Solvents were degassed by bubbling N_2 for at least 30 minutes prior to use. Reactions and filtrations were carried out under N_2 using standard Schlenk technique.

^{1}H NMR spectra were recorded in $CDCl_3$ or C_6D_6 at 298 K on Bruker 400 AVANCE and Bruker 300 AVANCE spectrometers fitted with a BBFO probe-head at 400 and 300 MHz respectively, using the solvent as internal standard (7.26 ppm for $CDCl_3$ and 7.16 ppm for C_6D_6).

^{13}C NMR spectra were recorded in $CDCl_3$ at 298 K on a Bruker 400 AVANCE spectrometer fitted with a BBFO probe-head at 101 MHz, using the solvent as internal standard (77.16 ppm).

^{31}P NMR spectra were recorded in $CDCl_3$ or C_6D_6 at 298 K on a Bruker 400 AVANCE spectrometer fitted with a BBFO probe-head at 162 MHz, using 85% H_3PO_4 as external standard (0 ppm).

^{19}F NMR spectra were recorded in $CDCl_3$ at 298 K on a Bruker 400 AVANCE spectrometer fitted with a BBFO probe-head at 376 MHz, using hexafluorobenzene as external standard (-164.9 ppm).

The terms m, s, d, t, q represent multiplet, singlet, doublet, triplet, quadruplet respectively, and the term br means a broad signal. Reported assignments are based on COSY, HSQC and H2BC correlation experiments.

Exact masses of complexes were recorded on a LTQ ORBITRAP XL Thermo Mass Spectrometer (electrospray source). Mass analysis on complexes were performed on an Infusion Water Acquity Ultra Performance LC HO6UPS-823M instrument (electrospray source, quadrupole analyser).

UV-Vis spectra were recorded on a Thermo Scientific Evolution 260 Bio UV Spectrophotometer.

IR spectra were collected with a Thermo Scientific Nicolet 5PCFT-IR-ATR spectrometer equipped with diamond crystal (3400-400 cm^{-1} interval).

2. Experimental procedures

Synthesis of Pd_2Ru complexes

$Pd(dba)_2$ (1 eq., 0.16 mmol, 92 mg), Ru_2(*p*-cymene)$_2Cl_4$ (1 eq., 0.16 mmol, 98 mg), sodium thiophenate (3 eq., 0.48 mmol, 64 mg) and the desired phosphine (1 eq. , 0.16 mmol) were sequentially added to a 50 ml Schlenk-type flask equipped with a magnetic stirring bar. The vessel underwent at least three vacuum/N_2 cycles and freshly degassed toluene (16 mL) was then syringed under N_2. The solution turned deep red within 10 minutes and $AgSbF_6$ (3 eq., 0.48 mmol, 164 mg) was then added under a nitrogen flux. The reaction mixture was stirred at room temperature and it gradually turned to a dark green color. Two hours later the crude was filtered under N_2 through a short pad of celite to remove traces of black metals. Toluene was evaporated under vacuum and the resulting solid was purified by flash column chromatography on silica gel using hexane/acetone or hexane/ethyl acetate as eluent. The green solid obtained was then triturated three times with pentane and dried under vacuum to afford a crystalline powder. Clusters were characterized by ^{1}H, ^{31}P, ^{19}F, ^{13}C NMR spectroscopy, HRMS or MS analysis, UV-Vis spectroscopy and IR spectrometry.

Synthesis of Pd(II) dimer A

$Pd(PPh_3)_4$ (1 eq., 0.17 mmol, 200 mg) and diphenyl disulfide (0.5 eq., 0.086 mmol, 19 mg) were added to a 50 ml Schlenk-type flask equipped with a magnetic stirring bar and the vessel underwent at least three vacuum/N_2 cycles. 20 ml of freshly degassed $CHCl_3$ were immediately syringed under N_2 and the resulting mixture quickly turned into a deep red solution. The crude mixture was kept under stirring at room temperature for 3 hours and the solvent was then removed under vacuum. The resulting solid was purified by $CHCl_3$/hexane washings (1/60 v/v, 3x30 ml). Evaporation of volatiles afforded a deep red solid. Crystals of the dimer were obtained upon crystallization by vapour diffusion using $CHCl_3$/hexane.

3. Extended screening of reaction conditions

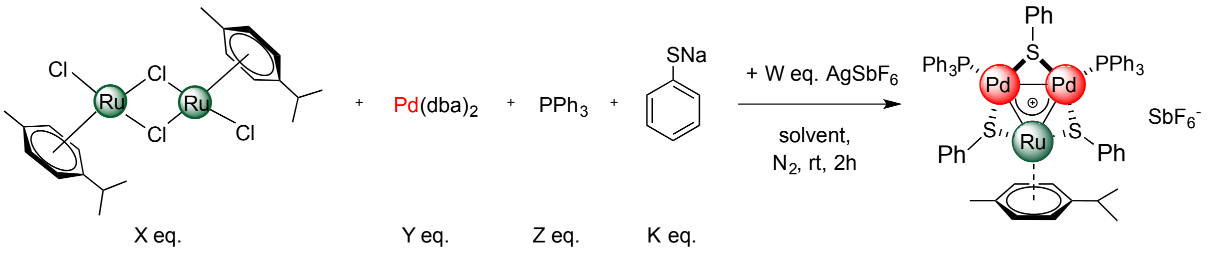

Main byproducts

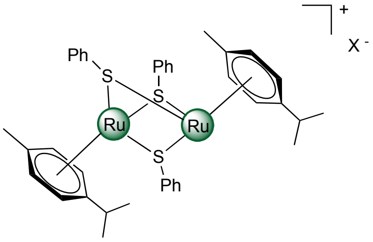

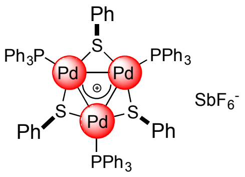

Effect of the solvent and concentration

Entry	Solvent	Conc. (M)	Yield (%)
1	toluene	0.01	26,[a] mixture with Ru(II) thiolate dimer
2	toluene	0.005	<10,[a] mixture with Ru(II) thiolate dimer and Pd_3^+
3	THF	0.01	<10,[a] mixture with Ru(II) thiolate dimer and Pd_3^+
4	CH_3CN	0.01	<10,[a] mixture with Ru(II) thiolate dimer and Pd_3^+

[a] ^{1}H NMR yield.

Effect of molar ratios

Entry	Ru	Pd	PPh_3	PhSNa	$AgSbF_6$	Yield (%)
1	1	1	1	3	3	26,[a] mixture with Ru(II) thiolate dimer
2	1	1	1	1	3	--
3	1	4	4	6	12	--
4	1	4	4	6	6	<10,[a] mixture with Pd_3^+
5	1	4	4	6	2	<10,[a] mixture with Pd_3^+

[a] ^{1}H NMR yield.

Effect of the thiolate source PhS-Y

Entry	Y	n	Yield (%)
1	Na^+	3	26,[a] mixture with with Ru(II) thiolate dimer
2	K^+	3	18,[a] mixture with with Ru(II) thiolate dimer
3	Ag^+	3	--
4	-SPh	1.5	--

[a] ^{1}H NMR yield.

Effect of the Ru and Pd precursor

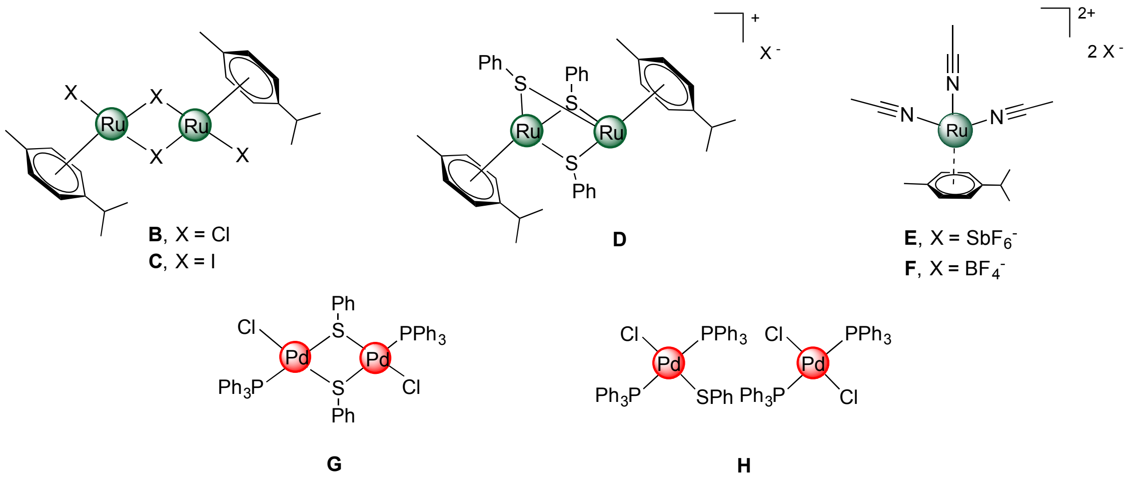

Entry	Ru / eq.	Pd / eq.	PPh_3	PhSNa	$AgSbF_6$	Yield (%)
1	B / 1	G / 1	--	1	2	--
2	B / 1	H / 1	--	--	2	--
3	C / 1	$Pd(dba)_2$ / 1	1	3	3	--
4	D / 1	$Pd(dba)_2$ / 2	2	--	2	--
5	E / 2	$Pd(dba)_2$ / 1	1	3	3	--
6	F / 1	$Pd(dba)_2$ / 1.3	1.3	4	4	--
7	F / 1	G / 1	--	1	2	--
8	F / 2	$Pd(dba)_2$ / 1	1	3	3	<10[a]
9	F / 3	$Pd(dba)_2$ / 1	1	3	3	<10[a]

[a] ^{1}H NMR yield.

4. MS analyses for heterobimetallic complexes

a) from 2 equiv. Pd(dba)$_2$ + 1 equiv. Pt(dba)$_3$

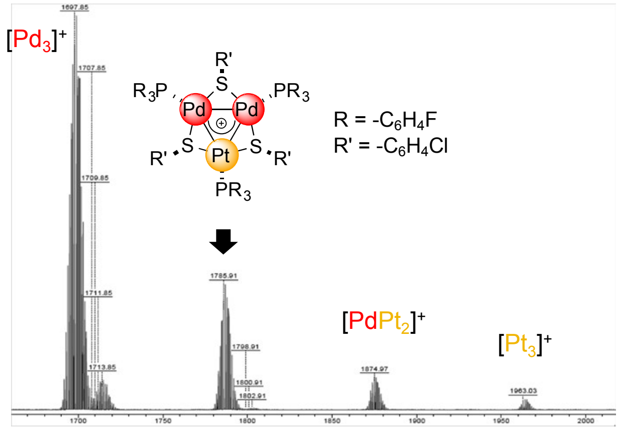

b) from 2 equiv. Pd(dba)$_2$ + 1 equiv. Pt(dba)$_3$

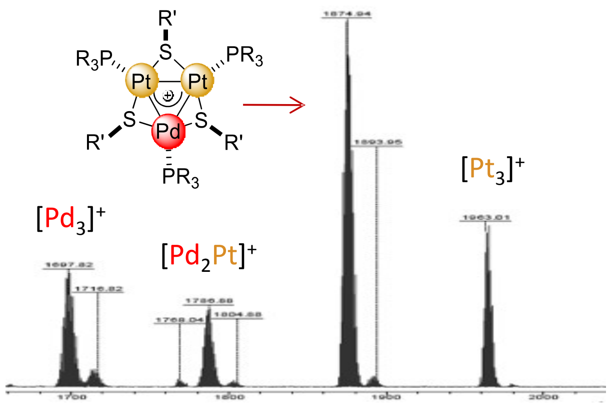

The reaction of zero-valent palladium and platinum precursors lead to the formation of a mixture of four trinuclear complexes, as mentioned in the main manuscript. The experiment can be monitored by MS analyses by ESI$^+$ thanks to the peculiar isotopic pattern of each cationic complex. This unselective process can be partially guided through the use of different ratios of substrates, although the two heterobimetallic species always formed together with the corresponding homonuclear ones. In particular, the reaction of Pd(dba)$_2$ (2 eq.) with Pt(dba)$_3$ (1 eq.) shows that the [Pd$_3$]$^+$ complex is the most abundant species (way a, above). Reversing the ratio of substrates, the heterobimetallic cluster [PdPt$_2$]$^+$ becomes the major product. In both cases, purification of each pure complex requires a series of column chromatography on silica gel (4 to 12). The combination of a discrete oxidized dimer with a reduced monomer mentioned in the main article could steer the selectivity of the whole process enabling to achieve higher yields of the desired heterobimetallic species.

5. Spectroscopic data of complexes

- Pd(II) dimer

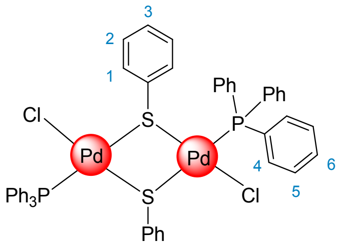

The complex was isolated as deep red crystals obtained upon crystallization by vapor diffusion using $CHCl_3$/hexane. Crystals of provided under ESI-*positive* analysis a main ion current deriving from the corresponding Pd_3^+ cation (centered at *m/z* 1433), which is likely generated during ionization. This suggests that the reactivity observed in solution can be exerted under MS conditions as well.

^{1}H NMR (400 MHz, C_6D_6) δ (ppm): 8.15 (d, *J*= 7.16 Hz, 4H, H_1), 7.60 (m, 12H, H_4), 6.84 (m, 24H, H_{2-3}, H_{5-6}).

^{31}P NMR (162 MHz, C_6D_6) δ (ppm): 30.23.

- Complex 1a

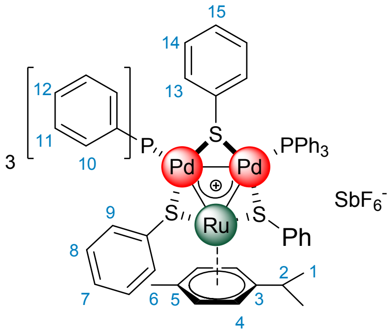

Isolated as deep green crystals obtained upon crystallization by vapour diffusion using CH_2Cl_2/hexane. Isolated yield = 13%. Owing to its reduced symmetry, efforts to collect a low-noise ^{13}C NMR spectrum were unsuccessful.

HRMS calculated for $C_{64}H_{59}P_2Pd_2RuS_3$ $[M]^+$: 1300.0400, found: 1300.0422.

^{1}H NMR (400 MHz, $CDCl_3$) δ (ppm): 7.35 (m, 18H, H_{11-12}), 7.18 (m, 6H, H_{10}), 7.11 (m, 6H, H_{10}), 6.98 (m, 4H, H_8), 6.93 (m, 4H, H_9), 6.85 (m, 2H, H_7), 6.68 (t, *J* = 6.9 Hz, 1H, H_{15}), 6.64 (d, *J* = 7.7 Hz, 2H, H_{13}), 6.42 (t, *J* = 7.9 Hz, 2H, H_{14}), 5.08 (d, *J* = 5.8 Hz, 1H, H_4), 4.84 (d, *J* = 4.9 Hz, 1H, H_4), 4.75 (d, *J* = 6.4 Hz, 1H, H_4), 4.55 (d, *J* = 6.4 Hz, 1H, H_4), 2.55 (m, 1H, H_2), 2.24 (s, 3H, H_6), 1.25 (m, 6H, H_1).

^{31}P NMR (162 MHz, CDCl$_3$) δ (ppm): 21.76 (d, J = 103.0 Hz, 1P), 19.98 (d, J = 103.0 Hz, 1P).

IR (ATR) ν (cm^{-1}): 3061, 2923, 2854, 1474, 1434, 1178, 1094, 1025, 999, 740, 689, 653, 520, 505.

UV-Vis (c = 2·10^{-5} M): λ_{max1} = 372 nm, ε_{max1} = 1.82·10^4 M^{-1}cm^{-1}; λ_{max2} = 423 nm, ε_{max2} = 1.64·10^4 M^{-1}cm^{-1}; λ_{max3} = 599 nm, ε_{max3} = 3.98·10^3 M^{-1}cm^{-1}.

Comparison between UV-Vis spectra of Pd$_3^+$ (left) and Pd$_2$Ru (right, samples concentration = 2·10^{-5} M)

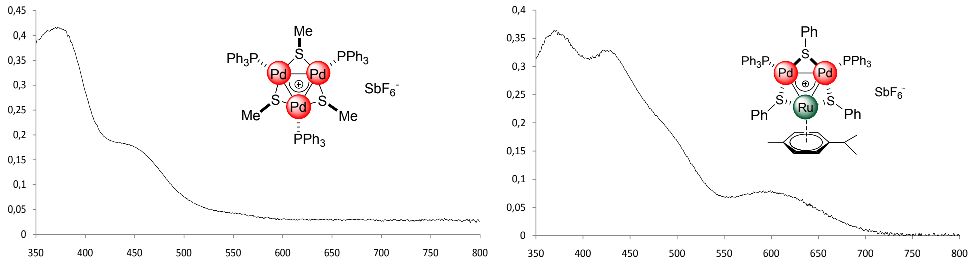

- Complex 1b

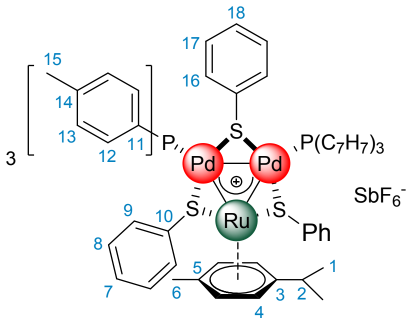

Isolated as deep green crystalline solid. Isolated yield = 32%.

HRMS calculated for $C_{70}H_{71}P_2Pd_2RuS_3$ [M]$^+$: 1384.1341, found: 1384.1384.

^{1}H NMR (400 MHz, CDCl$_3$) δ (ppm): 7.22 (dd, J_1= 10.8, J_2= 8.2 Hz, 6H, H$_{12}$), 7.06 (d, J_1= 11.0, J_2= 8.2 Hz, 6H, H$_{12}$), 7.01 (d, J = 7.8 Hz, 6H, H$_{13}$), 6.96 (m, 8H, H$_{8-9}$), 6.88 (d, J = 7.5 Hz, 6H, H$_{13}$), 6.83 (d, J = 7.6 Hz, 2H, H$_7$), 6.70 (t, J = 7.4 Hz, 1H, H$_{18}$), 6.63 (d, J = 7. 6 Hz, 2H, H$_{16}$), 6.42 (t, J = 7.6 Hz, 2H, H$_{17}$), 5.04 (d, J = 5.8 Hz, 1H, H$_4$), 4.85 (d, J = 6.0 Hz, 1H, H$_4$), 4.65 (d, J = 5.7 Hz, 1H, H$_4$), 4.53 (d, J = 5.9 Hz, 1H, H$_4$), 2.58 (dt, J_1= 13.5, J_2= 6.7 Hz, 1H, H$_2$), 2.31 (s, 9H, H$_{15}$), 2.27 (s, 9H, H$_{15}$), 2.22 (s, 3H, H$_6$), 1.25 (m, 6H, H$_1$).

^{13}C NMR (101 MHz, CDCl$_3$) δ (ppm): 141.0 (C$_{14}$), 140.6 (C$_{14}$), 138.3 (C$_{10}$), 136.3 (C$_{10}$), 133.6 (d, $^2J_{CP}$ = 12.2 Hz, C$_{12}$), 133.4 (d, $^2J_{CP}$ = 12.5 Hz, C$_{12}$), 133.1 (C$_{7or8or9}$), 132.7 (C$_{7or8or9}$), 132.4 (C$_{7or8or9}$), 129.4 (d, $^3J_{CP}$ = 10.6 Hz, C$_{13}$), 129.0 (d, $^3J_{CP}$ = 10.2 Hz, C$_{13}$), 127.3-126.8 (C$_{11}$), 107.9 (C$_{3or5}$), 100.6 (C$_{3or5}$), 87.7 (C$_4$), 86.8 (C$_4$), 85.4 (C$_4$), 81.7 (C$_4$), 53.17, 30.8 (C$_2$) , 24.2 (C$_1$), 33.3 (C$_1$), 21.5 (C$_{15}$), 21.3 (C$_6$).

^{31}P NMR (162 MHz, $CDCl_3$) δ (ppm): 20.37 (d, J = 103.2 Hz, 1P), 18.47 (d, J = 103.0 Hz, 1P).

IR (ATR) ν (cm^{-1}): 2967, 2918, 2872, 1696, 1405, 1095, 1021, 855, 744, 657, 521.

UV-Vis (c = 2·10^{-5}M, THF): λ_{max1} = 368 nm, ε_{max1} = 6.45·10^3 $M^{-1}cm^{-1}$; λ_{max2} = 428 nm, ε_{max2} = 5.65·10^3 $M^{-1}cm^{-1}$; λ_{max3} = 581 nm, ε_{max3} = 1.28·10^3 $M^{-1}cm^{-1}$.

- Complex 1c

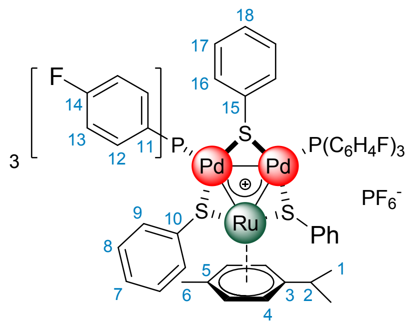

Isolated as deep green crystalline solid. Isolated yield = 16%.

HRMS calculated for $C_{64}H_{53}F_6P_2Pd_2RuS_3$ $[M]^+$: 1407.9835, found: 1407.9857.

^{1}H NMR (400 MHz, $CDCl_3$) δ (ppm): 7.28 (m, 6H, H_{13}), 7.15 (m, 6H, H_{13}), 7.00 (t, J = 7.8 Hz, 4H, H_8), 6.96 (m, 12H, $H_{7,9,12}$), 6.81 (t, J = 8.2 Hz, 6H, H_{12}), 6.76 (d, J = 7.4 Hz, 1H, H_{18}), 6.67 (d, J = 7.4 Hz, 2H, H_{16}), 6.50 (t, J = 7.7 Hz, 2H, H_{17}), 5.42 (d, J = 5.9 Hz, 1H, H_4), 5.30 (d, J = 6.0 Hz, 1H, H_4), 4.83 (d, J = 6.1 Hz, 1H, H_4), 4.48 (m, J = 6.1 Hz, 1H, H_4), 2.40 (m, 1H, H_2), 2.24 (s, 3H, H_6), 1.27 (d, J = 6.8 Hz, 3H, H_1), 1.19 (d, J = 6.8 Hz, 3H, H_1).

^{13}C NMR (101 MHz, $CDCl_3$) δ (ppm): 164.1 (d, $^1J_{CF}$ = 254 Hz, C_{14}), 163.9 (d, $^1J_{CF}$ = 252 Hz, C_{14}), 140.8 (C_{10or15}), 138.3 (C_{10or15}), 136.3 (m, C_{12}), 133.3 ($C_{7\text{-}9or16\text{-}18}$), 132.7 ($C_{7\text{-}9or16\text{-}18}$), 132.6 ($C_{7\text{-}9or16\text{-}18}$), 131.8 ($C_{7\text{-}9or16\text{-}18}$), 128.7 ($C_{7\text{-}9or16\text{-}18}$), 127.9 ($C_{7\text{-}9or16\text{-}18}$), 127.3 (d, J_{CP} = 6.6 Hz, C_{11}), 116.3 (d, $^2J_{CF}$ = 21.2 Hz, C_{13}), 116.2 (d, $^2J_{CF}$ = 21.2 Hz, C_{13}), 115.8 (d, $^2J_{CF}$ = 21.4 Hz, C_{13}), 115.7 (d, $^2J_{CF}$ = 21.5 Hz, C_{13}), 107.7 (C_{3or5}), 102.0 (C_{3or5}), 90.7 (C_4), 87.0 (C_4) , 85.6 (C_4), 83.2 (C_4), 31.8 (C_2), 23.2 (C_1), 23.0 (C_1), 21.5 (C_6).

^{31}P NMR (162 MHz, $CDCl_3$) δ (ppm): 17.72 (d, J = 105.0 Hz, 1P), 16.64 (d, J = 105.8 Hz, 1P), -144.17 (apparent quintet, J_{PF} = 718.8 Hz, PF_6).

^{19}F NMR (377 MHz, $CDCl_3$) δ (ppm): -71.81, -73.70, -107.83, -108.61.

IR (ATR) ν (cm^{-1}): 2953, 2918, 2856, 1586, 1495, 1438, 1394, 1231, 1161, 1092, 827, 742, 691, 556, 527, 447.

UV-Vis (c = 2·10^{-5}M, THF): λ_{max1} = 368 nm, ε_{max1} = 1.23·10^4 $M^{-1}cm^{-1}$; λ_{max2} = 411 nm, ε_{max2} = 8.45·10^3 $M^{-1}cm^{-1}$; λ_{max3} = 604 nm, ε_{max3} = 1.47·10^3 $M^{-1}cm^{-1}$.

- Complex 1d

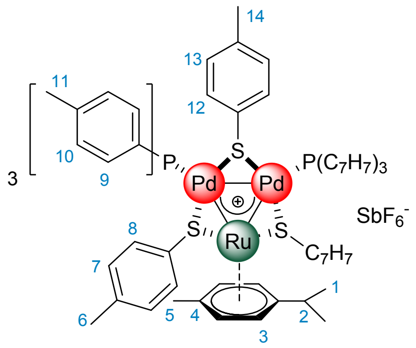

Isolated as deep green crystalline solid. Isolated yield = 6%. Owing to its reduced symmetry, efforts to collect a low-noise ^{13}C NMR spectrum were unsuccessful.

MS calculated for $C_{73}H_{77}P_2Pd_2RuS_3$ $[M]^+$: 1425.1770, found: 1426.795.

^1H NMR (400 MHz, $CDCl_3$) δ (ppm): 7.16 (dd, J_1= 10.8, J_2= 8.1 Hz, 6H, H_9), 7.07 (dd, J_1= 10.8, J_2= 8.1 Hz, 6H, H_9), 6.99 (d, J = 7.2 Hz, 6H, H_{10}), 6.88 (d, J = 7.3 Hz, 6H, H_{10}), 6.82 (m, 4H, H_7), 6.76 (d, J = 8.0 Hz, 2H, H_8), 6.63 (d, J = 7.8 Hz, 2H, H_8), 6.50 (d, J = 7.9 Hz, 2H, H_{13}), 6.20 (d, J = 7.9 Hz, 2H, H_{12}), 4.99 (d, J = 5.8 Hz, 1H, H_3), 4.83 (d, J = 5.8 Hz, 1H, H_3), 4.59 (d, J = 5.7 Hz, 1H, H_3), 4.54 (d, J = 5.7 Hz, 1H, H_3), 2.62 (m, 1H, H_2), 2.31 (s, 9H, H_{11}), 2.29 (s, 9H, H_{11}), 1.28 (m, 6H, H_1).

^{31}P NMR (162 MHz, $CDCl_3$) δ (ppm): 20.32 (d, J = 101.3 Hz, 1P), 18.54 (d, J = 101.5 Hz, 1P).

IR (ATR) ν (cm^{-1}): 2926, 2848, 1602, 1471, 1397, 1094, 1010, 801, 655, 549, 498.

UV-Vis (c = $2\cdot10^{-5}$M, THF): λ_{max1} = 377 nm, ε_{max1} = $2.17\cdot10^4$ $M^{-1}cm^{-1}$; λ_{max2} = 412 nm, ε_{max2} = $1.96\cdot10^4$ $M^{-1}cm^{-1}$; λ_{max3} = 581 nm, ε_{max3} = $4.28\cdot10^3$ $M^{-1}cm^{-1}$.

- Complex 1e

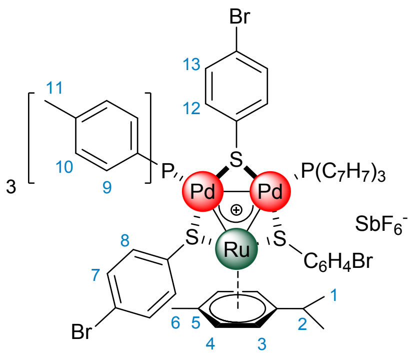

Isolated as deep green crystalline solid. Isolated yield = = 20%. Owing to its reduced symmetry, efforts to collect a low-noise ^{13}C NMR spectrum were unsuccessful.

MS calculated for $C_{70}H_{68}Br_3P_2Pd_2RuS_3$ $[M]^+$: 1620.838, found: 1620.417.

^1H NMR (400 MHz, $CDCl_3$) δ (ppm): 7.13 (dd, J_1= 11.0, J_2= 8.1 Hz, 6H, H_9), 7.05 (m,16H, H_{9-10}, H_7), 6.98 (d, J = 6.6 Hz, 4H, H_8), 6.94 (d, J = 8.0 Hz, 6H, H_{10}), 6.81 (d, J = 8.3 Hz, 2H, H_7), 6.75 (d, J = 8.3 Hz, 2H, H_8), 5.26 (d, J = 6.0 Hz, 1H, H_{3-4}), 4.91 (d, J = 6.1 Hz, 1H, H_{3-4}), 4.88 (d, J = 6.0 Hz, 1H, H_{3-4}), 4.55 (d, J = 5.8 Hz, 1H, H_{3-4}), 2.56 (m, 1H, H_2), 2.33 (s, 18H, H_{11}), 2.28 (s, 3H, H_6), 1.29 (d, J = 6.8 Hz, 3H, H_1), 1.24 (m, 3H, H_1).

^{31}P NMR (162 MHz, $CDCl_3$) δ (ppm): 20.64 (d, J = 100.8 Hz, 1P), 18.76 (d, J = 100.5 Hz, 1P).

IR (ATR) ν (cm^{-1}): 2926, 2852, 1465, 1375, 1260, 1093, 1005, 801, 707, 654, 629, 610, 518, 489, 418.

UV-Vis (c = 2·10^{-5} M, THF): λ_{max1} = 370 nm, ε_{max1} = 1.74·10^4 $M^{-1}cm^{-1}$; λ_{max2} = 431 nm, ε_{max2} = 1.38·10^4 $M^{-1}cm^{-1}$; λ_{max3} = 597 nm, ε_{max3} = 3.38·10^3 $M^{-1}cm^{-1}$.

- Complex 1f

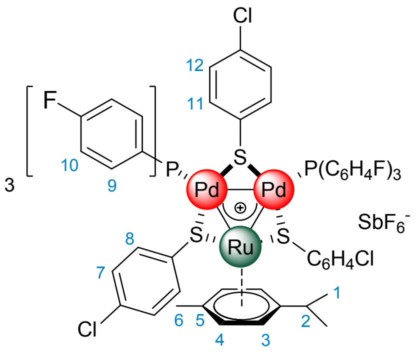

Isolated as deep green crystalline solid. Isolated yield = 14%. Owing to its reduced symmetry, efforts to collect a low-noise ^{13}C NMR spectrum were unsuccessful.

MS calculated for $C_{64}H_{50}Cl_3F_6P_2Pd_2RuS_3$ $[M]^+$: 1511.464, found: 1512.380.

^{1}**H NMR (400 MHz, CDCl$_3$) δ (ppm):** 7.26 (m, 6H, H_{10}), 7.18 (m, 6H, H_{10}), 7.02 (t, J = 8.0 Hz, 6H, H_9 + 4H, H_7), 6.96 (m, 6H, $H_{7\text{-}8}$), 6.90 (t, J = 8.4 Hz, 6H, H_9), 6.85 (d, J = 8.3 Hz, 2H, H_8), 6.59 (d, J = 8.3 Hz, 2H, H_{12}), 6.46 (d, J = 8.3 Hz, 2H, H_{11}), 5.52 (d, J = 6.1 Hz, 1H, $H_{3\text{-}4}$), 5.39 (d, J = 6.0 Hz, 1H, $H_{3\text{-}4}$), 4.87 (d, J = 6.1 Hz, 1H, $H_{3\text{-}4}$), 4.54 (m, J = 6.1 Hz, 1H, $H_{3\text{-}4}$), 2.44 (m, 1H, H_2), 2.28 (s, 3H, H_6), 1.29 (d, J = 6.9 Hz, 3H, H_1), 1.22 (d, J = 6.9 Hz, 3H, H_1).

^{31}**P NMR (162 MHz, CDCl$_3$) δ (ppm):** 17.59 (d, J = 104.2 Hz, 1P), 16.78 (d, J = 103.8 Hz, 1P).

^{19}**F NMR (377 MHz, CDCl$_3$) δ (ppm):** -107.05, -107.61.

IR (ATR) ν (cm^{-1}): 2921, 1588, 1495, 1470, 1392, 1231, 1160, 1088, 1010, 825, 658, 527, 495, 488, 449, 440.

UV-Vis (c = 2·10^{-5} M, THF): λ_{max1} = 377 nm, ε_{max1} = 1.90·10^4 M^{-1}cm^{-1}; λ_{max2} = 427 nm, ε_{max2} = 1.58·10^4 M^{-1}cm^{-1}; λ_{max3} = 596 nm, ε_{max3} = 3.23·10^3 M^{-1}cm^{-1}.

- Complex 1g

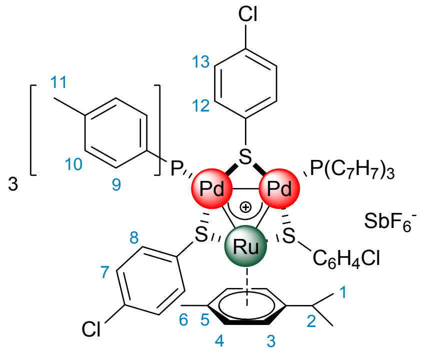

Isolated as deep green crystalline solid. Isolated yield = 36%. Owing to its reduced symmetry, a few 13C resonances were not detected in the corresponding spectrum.

MS calculated for $C_{70}H_{68}Cl_3P_2Pd_2RuS_3$ [M]$^+$: 1487.046, found: 1487.591.

^{1}**H NMR (400 MHz, CDCl$_3$) δ (ppm):** 7.14 (d, J_1= 11.0, J_2= 8.0 Hz, 6H, H_9), 7.06 (dd, J_1= 11.0, J_2= 8.0 Hz, 6H, H_9), 7.02 (d, J = 9.0 Hz, 6H, H_{10}), 6.93 (m, 8H, H_{10}, H_7), 6.88 (d, J = 8.4 Hz, 2H, H_7), 6.81 (m, 4H, H_8), 6.51 (d, J = 8.5 Hz, 2H, H_{13}), 6.29 (d, J = 8.5 Hz, 2H, H_{12}), 5.26 (d, J = 6.0 Hz, 1H, $H_{3\text{-}4}$), 4.90 (d, J = 6.4 Hz, 2H, $H_{3\text{-}4}$), 4.55 (d, J = 5.9 Hz, 1H, $H_{3\text{-}4}$), 2.56 (m, 1H, H_2), 2.32 (s, 18H, H_{11}), 2.28 (s, 3H, H_6), 2.56 (m, 1H, H_2), 1.29 (d, J = 6.9 Hz, 3H, H_1), 1.24 (d, J = 7.0 Hz, 3H, H_1).

^{31}**P NMR (162 MHz, CDCl$_3$) δ (ppm):** 20.68 (d, J = 100.6 Hz, 1P), 18.79 (d, J = 100.7 Hz, 1P).

^{13}**C NMR (101 MHz, CDCl$_3$) δ (ppm):** 141.1 (C_{11}), 140.9 (C_{11}), 134.3 (C_{7or8}), 133.9 (C_{7or8}), 133.5 (d, $^2J_{CP}$ = 12.2 Hz, C_9), 133.4 (d, $^2J_{CP}$ = 12.5 Hz, C_9), 130.4 (C_{8a}), 129.3 (d, $^3J_{CP}$ = 10.6 Hz, C_{10}), 128.9 (d, $^3J_{CP}$ = 10.2 Hz, C_{10}), 128.5-127.9 (C_{9a}), 127.8, 127.1, 88.1 (C_4), 82.3 (C_3), 53.8, 31.6 (C_2), 29.7 (C_1), 29.3 (C_1).

IR (ATR) ν (cm^{-1}): 2962, 2919, 2857, 1595, 1470, 1392, 1092, 1011, 801, 658, 516.

UV-Vis (c = 2·10^{-5}M, THF): λ_{max1} = 371 nm, ε_{max1} = 2.04·10^{4} M^{-1}cm^{-1}; λ_{max2} = 432 nm, ε_{max2} = 1.73·10^{4} M^{-1}cm^{-1}; λ_{max3} = 588 nm, ε_{max3} = 4.26·10^{3} M^{-1}cm^{-1}.

6. Summary of crystallographic data

Data collection was performed at the X-ray diffraction beamline (XRD1) of the Elettra Synchrotron, Trieste (Italy)[1] at 100 K (nitrogen stream supplied through an Oxford Cryostream 700). Crystals were dipped in NHV oil (Jena Bioscience GmbH) and mounted on the goniometer head with a cryoloop. Complete datasets have been obtained merging two different data collections done on the same crystal, mounted with different orientations. Data were acquired using a monochromatic wavelength of 0.700 Å on a Pilatus 2M hybrid-pixel area detector. The diffraction data were indexed and integrated using XDS.[2] Scaling have been done using CCP4-Aimless code.[3, 4] The structures was solved by the dual space algorithm implemented in the SHELXT code.[5] Fourier analysis and refinement were performed by the full-matrix least-squares methods using SHELXL-2014[6], implemented in Olex2[7].

Compound **1a** was solved and refined in the centrosymmetric triclinic P-1 space group (R_{int}: 2.3%, R_1: 5.4%). All the non-H atoms were refined with anisotropic displacement parameters. The asymmetric unit includes one [Pd_2Ru(p-cymene)$(PPh_3)_2(SPh)_3$] molecule, half molecule of chloroform of crystallization and the SbF_6 anion, which was found located over two distinct sites. The first one was disordered over two positions, with Sb atom lying on an inversion center. The three fluorine atoms of each part were refined with 0.35 and 0.65 site occupancy factors, respectively. The second SbF_6 anion was overlaid with the chloroform molecule and was modelled with a site occupancy factor of 0.5. This confirms that the overall stoichiometry corresponds to one $SbF6^-$ anion (2 x 0.5) for each molecule of heterometallic trinuclear cluster. CDC 2017729 contains the crystallographic data for complex **1a**.

Crystal data and structure refinement

Identification code	am230v-bis_1_	
Empirical formula	C64 H59 P2 Pd2 Ru S3, 2(F3 Sb0.5), 0.5(C Cl3)	
Formula weight	1595.03	
Temperature	100 K	
Wavelength	0.700 Å	
Crystal system	Triclinic	
Space group	P -1	
Unit cell dimensions	a = 11.9921(3) Å	α= 94.5180(16)°.
	b = 12.28439(18) Å	β= 91.0356(18)°.
	c = 21.7330(5) Å	γ = 101.5240(16)°.
Volume	3125.45(11) $Å^3$	
Z	2	
Density (calculated)	1.695 Mg/m^3	
Absorption coefficient	1.467 mm^{-1}	
F(000)	1581	
Crystal size	0.15 x 0.10 x 0.10 mm^3	
Theta range for data collection	1.7060 to 32.8820°	
Index ranges	-14<=h<=14, -15<=k<=15, -27<=l<=27	
Reflections collected	35985	
Independent reflections	11869 [R(int) = 0.0152]	
Max. and min. transmission	1.00000 and 0.69180	
Refinement method	Full-matrix least-squares on F2	
Data / restraints / parameters	11869 / 316 / 803	
Goodness-of-fit on F2	1.329	
Final R indices [I>2sigma(I)]	R1 = 0.0504, wR2 = 0.1275	
R indices (all data)	R1 = 0.0515, wR2 = 0.1279	
Extinction coefficient	n/a	

Atomic coordinates (x 10^4) and equivalent isotropic displacement parameters ($Å^2$ x 10^3). U(eq) is defined as one third of the trace of the orthogonalized U^{ij} tensor

	x	y	z	U(eq)	
Pd2	6281.7(4)	4851.3(4)	2186.2(2)	13(1)	
Pd1	6788.1(4)	3155.2(4)	2814.2(2)	14(2)	
Ru1	8473.4(4)	4450.5(4)	2203.3(2)	14(2)	
S2	7352.1(13)	4587.7(13)	1342.1(7)	15(2)	
S3	7917.5(13)	2525.5(13)	2101.8(7)	16(1)	
S1	6011.6(13)	4622.9(14)	3225.1(7)	17(1)	
P2	4993.7(13)	5849.4(14)	1871.3(7)	14(2)	
P1	5757.4(14)	1573.6(14)	3165.4(7)	16(1)	
C12	4241(5)	6412(5)	2503(3)	15(2)	
C132	3929(5)	5067(5)	1310(3)	15(1)	
C14	6797(6)	3315(6)	882(3)	18(1)	
C64	7559(6)	2738(6)	601(3)	20(2)	
C22	4910(5)	7074(6)	2973(3)	18(2)	C182
3187(6)	4122(6)	1481(3)	21(2)		
C32	4405(6)	7567(6)	3460(3)	21(2)	
C26	1011.1(5)	4550(6)	2697(3)	23(2)	
C71	6496(5)	442(5)	3273(3)	19(2)	
C65	9134(6)	1806(6)	3049(3)	23(2)	
C161	2939(6)	-27(6)	1721(3)	25(1)	
C72	5632(5)	7119(5)	1515(3)	16(2)	
C11	5094(6)	1808(6)	3895(3)	22(1)	
C36	1032.7(5)	4931(6)	2101(3)	23(2)	
C82	5069(6)	7999(6)	1506(3)	19(1)	
C24	5637(6)	2992(6)	743(3)	20(2)	
C34	5251(6)	2066(6)	333(3)	24(2)	
C141	4483(6)	1477(6)	2069(3)	21(1)	
C62	3064(5)	6250(6)	2532(3)	22(2)	
C13	4582(6)	4283(6)	3432(3)	24(1)	
C42	3255(6)	7397(6)	3491(3)	25(1)	
C81	6810(6)	160(6)	3843(3)	27(2)	
C142	3928(6)	5305(6)	694(3)	24(2)	
C16	9428(6)	5023(6)	3116(3)	22(1)	
C46	9855(5)	5826(6)	1915(3)	22(2)	

C181	3929(6)	-91(6)	2680(3)	24(1)
C172	2458(6)	3421(6)	1048(3)	25(1)
C151	3622(6)	974(6)	1633(3)	24(2)
C52	2557(6)	6730(6)	3023(3)	26(2)
C76	9257(7)	4668(7)	3764(3)	33(2)
C15	8959(5)	1864(6)	2421(3)	19(1)
C63	4258(7)	4740(7)	4000(4)	34(2)
C131	4632(5)	937(6)	2595(3)	19(2)
C54	7156(6)	1815(6)	193(3)	25(1)
C33	2624(6)	3346(7)	3249(4)	29(2)
C112	7237(6)	8233(6)	1048(3)	28(1)
C92	5584(6)	8991(6)	1274(3)	22(1)
C23	3732(6)	3567(6)	3061(3)	27(1)
C86	1005.0(6)	6296(7)	1295(3)	32(2)
C122	6720(6)	7232(6)	1282(3)	24(1)
C162	2481(7)	3652(7)	437(4)	33(1)
C171	3085(6)	-568(6)	2247(3)	25(2)
C21	5790(6)	2433(7)	4365(3)	29(1)
C25	9563(6)	1322(7)	1998(4)	31(1)
C44	6000(7)	1466(6)	60(3)	26(1)
C35	1035.2(7)	735(8)	2210(5)	45(1)
C121	6848(6)	108(6)	2734(3)	25(2)
C111	7492(6)	-894(6)	2786(4)	27(2)
C102	6672(6)	9108(6)	1051(3)	25(2)
C55	9919(6)	1224(7)	3258(4)	33(1)
C56	9133(6)	6282(6)	2331(3)	24(1)
C91	7484(7)	-625(7)	3885(4)	33(2)
C61	3959(6)	1454(8)	4001(3)	33(2)
C152	3222(7)	4598(7)	261(3)	32(1)
C66	8892(6)	5875(6)	2913(3)	22(1)
C101	7837(6)	-1156(6)	3356(4)	30(2)
C43	2327(7)	3799(8)	3802(4)	38(1)
C51	3522(7)	1716(9)	4568(4)	44(1)
C31	5364(7)	2683(8)	4931(4)	38(2)
C45	1053.6(7)	693(7)	2830(5)	41(2)
C96	1089.7(7)	7411(8)	1387(4)	39(2)
C53	3155(8)	4484(8)	4178(4)	45(2)
C106	1046.5(9)	5524(9)	808(4)	51(2)

C41	4209(7)	2331(8)	5031(4)	38(2)
Sb1	1000.0	0	0	24(2)
F0AA	1158.4(6)	434(7)	155(5)	38(2)
F1AA	9819(7)	1401(7)	320(5)	52(3)
F3	9864(8)	-523(9)	780(4)	56(3)
Sb2	424.5(12)	8083.0(17)	4662.3(6)	44(1)
F52	1066(13)	6863(12)	4369(11)	157(9)
F22	496(19)	759(2)0	5428(7)	166(9)
F32	-175(15)	9204(14)	4973(13)	155(8)
F62	1881(9)	8838(12)	4786(7)	86(4)
F72	-1044(13)	736(2)0	4512(12)	118(8)
F42	42(2)0	866(4)0	3935(13)	212(12)
F3A	9055(13)	300(14)	618(7)	57(5)
F1A	1125.2(14)	286(14)	552(8)	53(5)
F2A	1026.9(12)	1515(11)	-173(8)	47(4)
Cl5	1454(5)	7808(7)	5049(3)	78(2)
Cl1	683(7)	8436(9)	3882(3)	78(3)
Cl3	-949(6)	7475(8)	4777(4)	71(2)
C5	445(17)	824(4)0	4673(4)	90(2)

7. Copies of NMR spectra

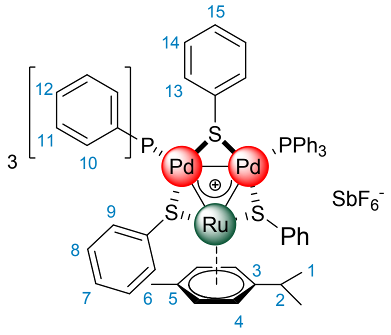

^{1}H NMR

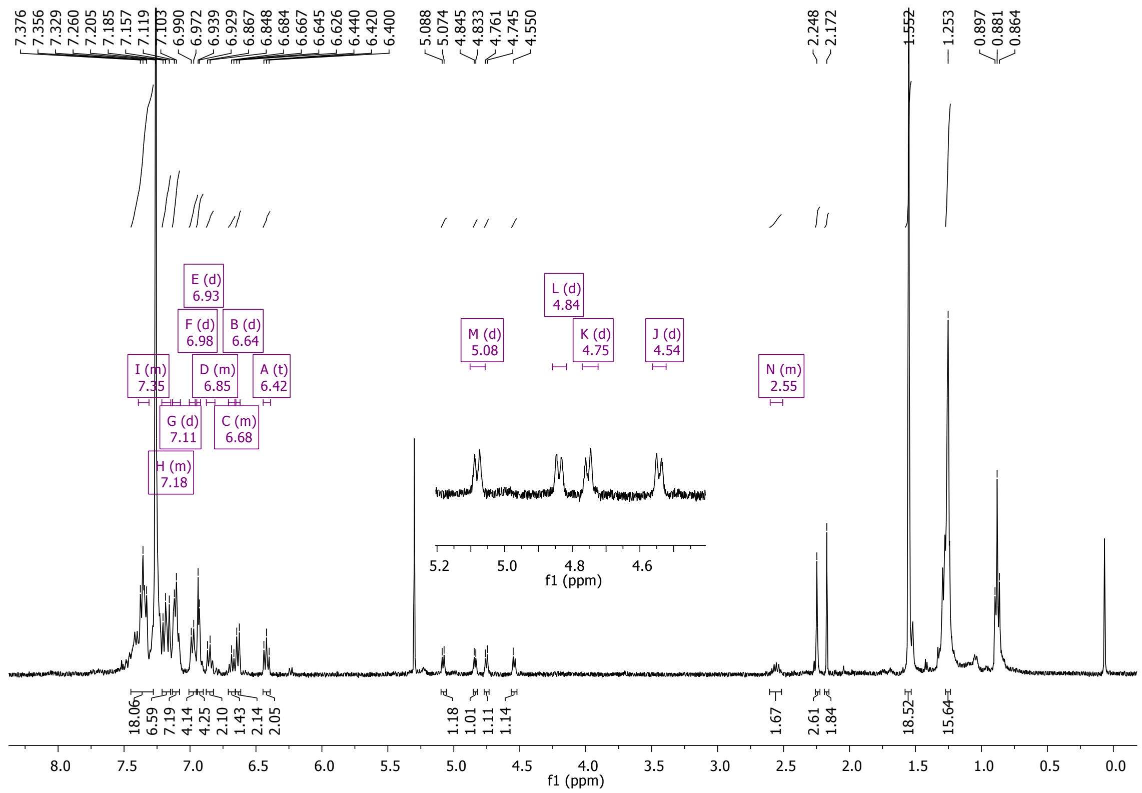

^{31}P NMR

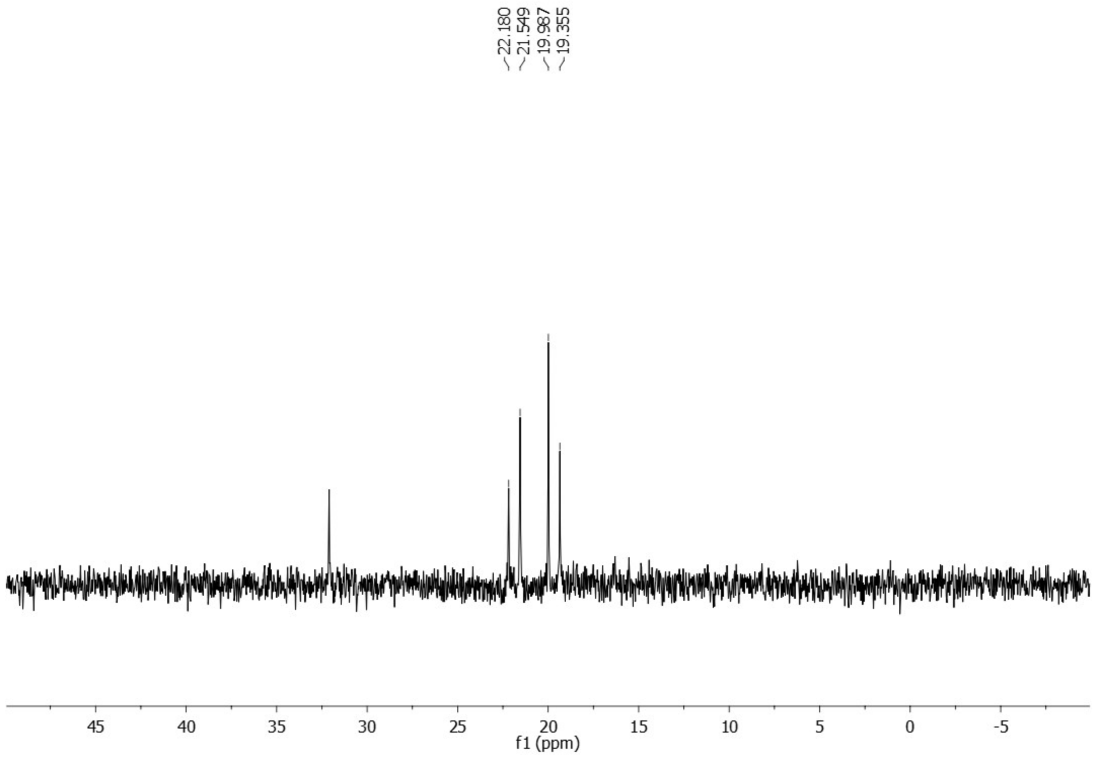

^{13}C NMR (12 Kscans)

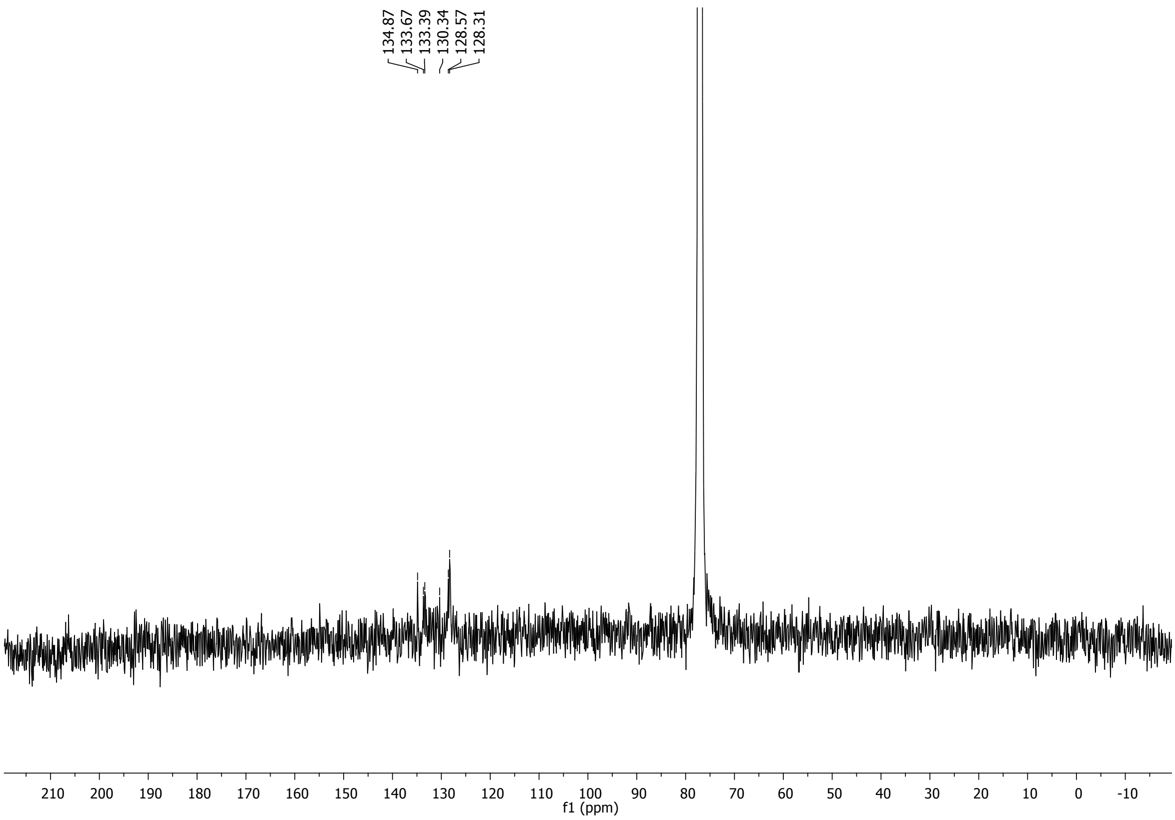

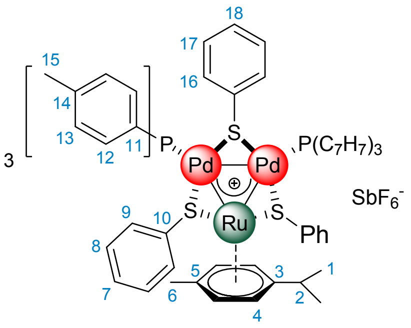

¹H NMR

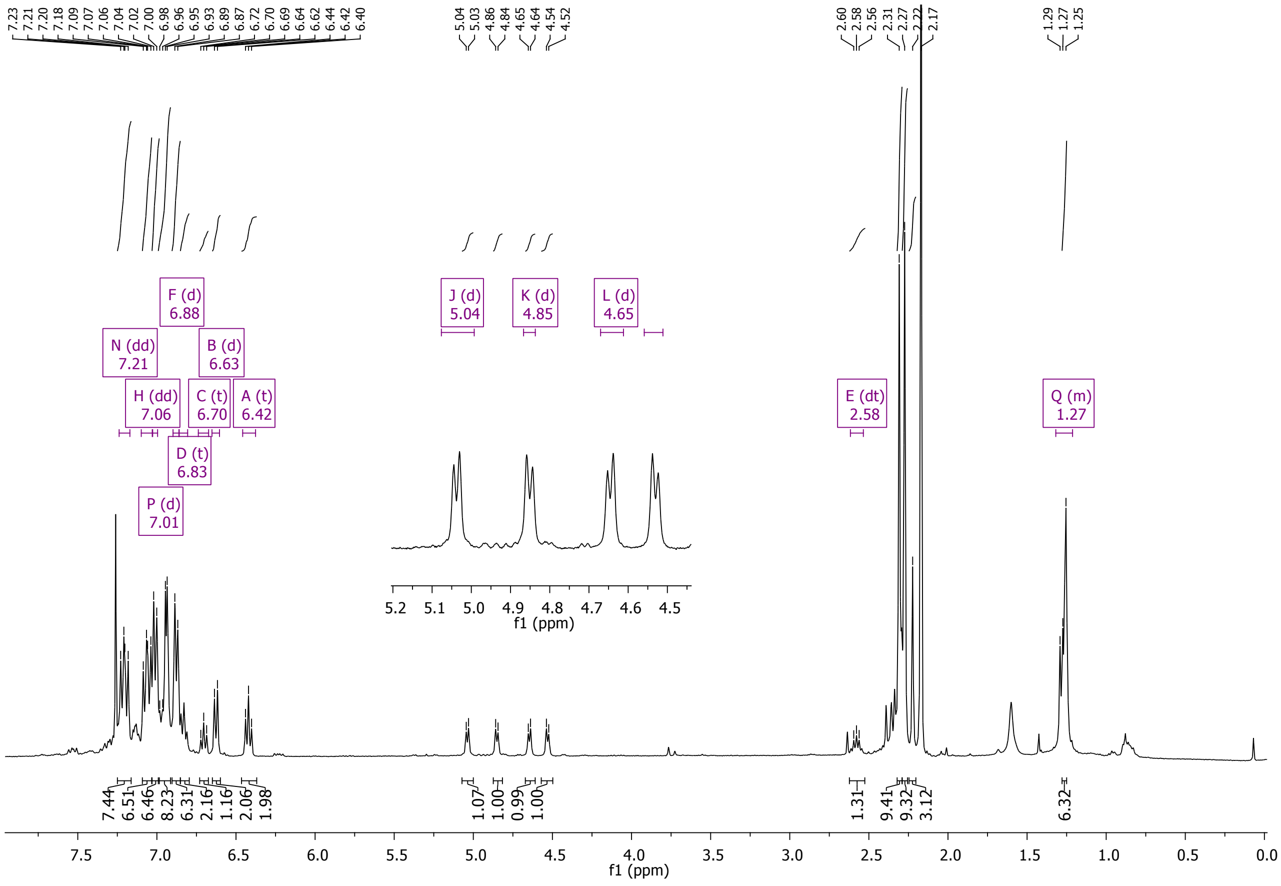

^{31}P NMR

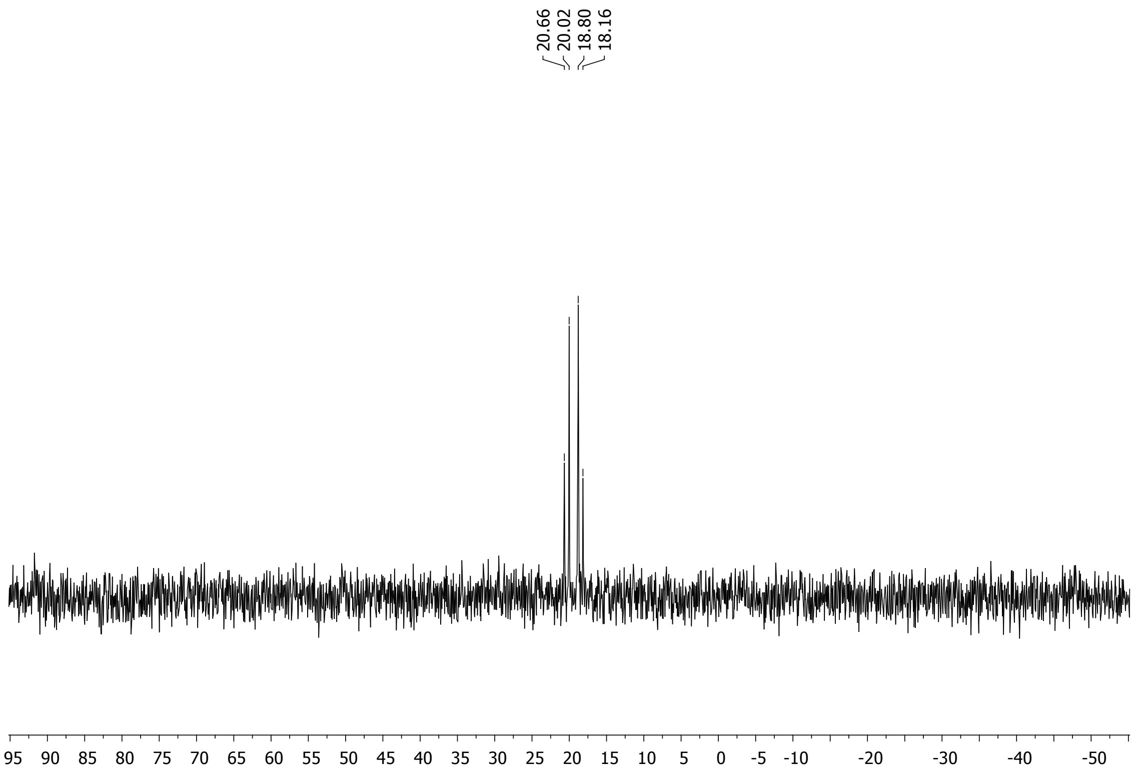

^{13}C NMR

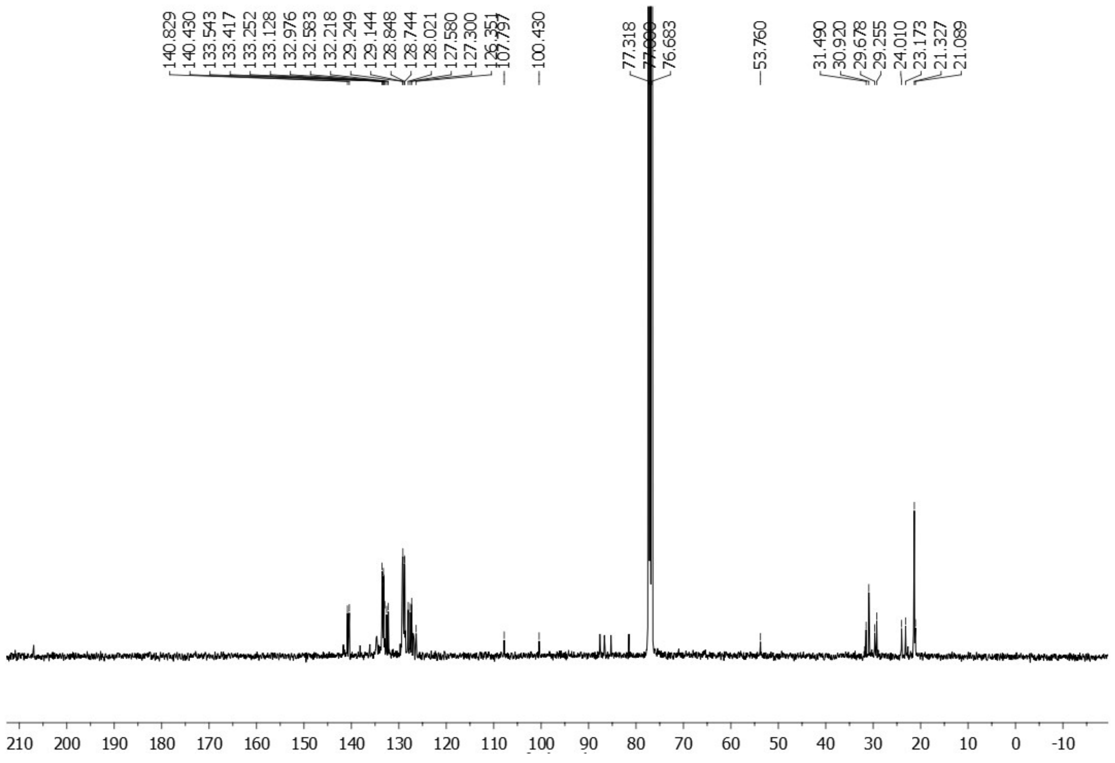

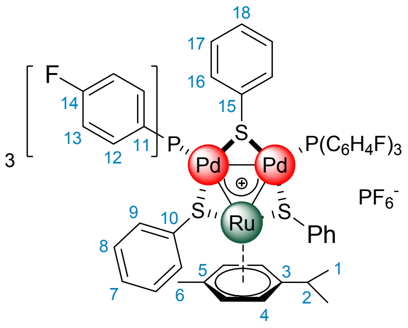
3
F
14
13
12
11
P
18
17
16
15
S
Pd
Pd
P(C6H4F)3
PF6-
9
10
S
Ru
S
Ph
8
7
6
5
4
3
2
1

^{1}H NMR

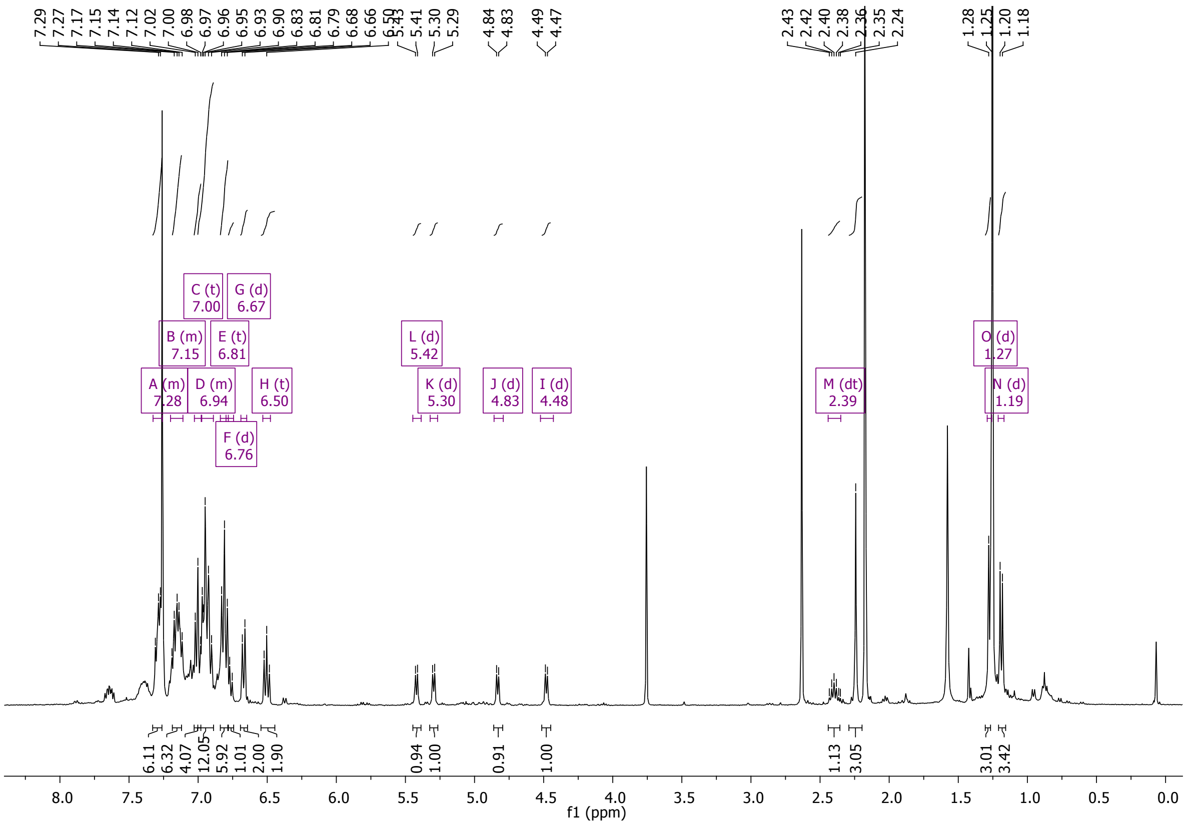
A (m)
7.28
B (m)
7.15
C (t)
7.00
D (m)
6.94
E (t)
6.81
F (d)
6.76
G (d)
6.67
H (t)
6.50
I (d)
4.48
J (d)
4.83
K (d)
5.30
L (d)
5.42
M (dt)
2.39
N (d)
1.19
O (d)
1.27
f1 (ppm)

^{31}P NMR

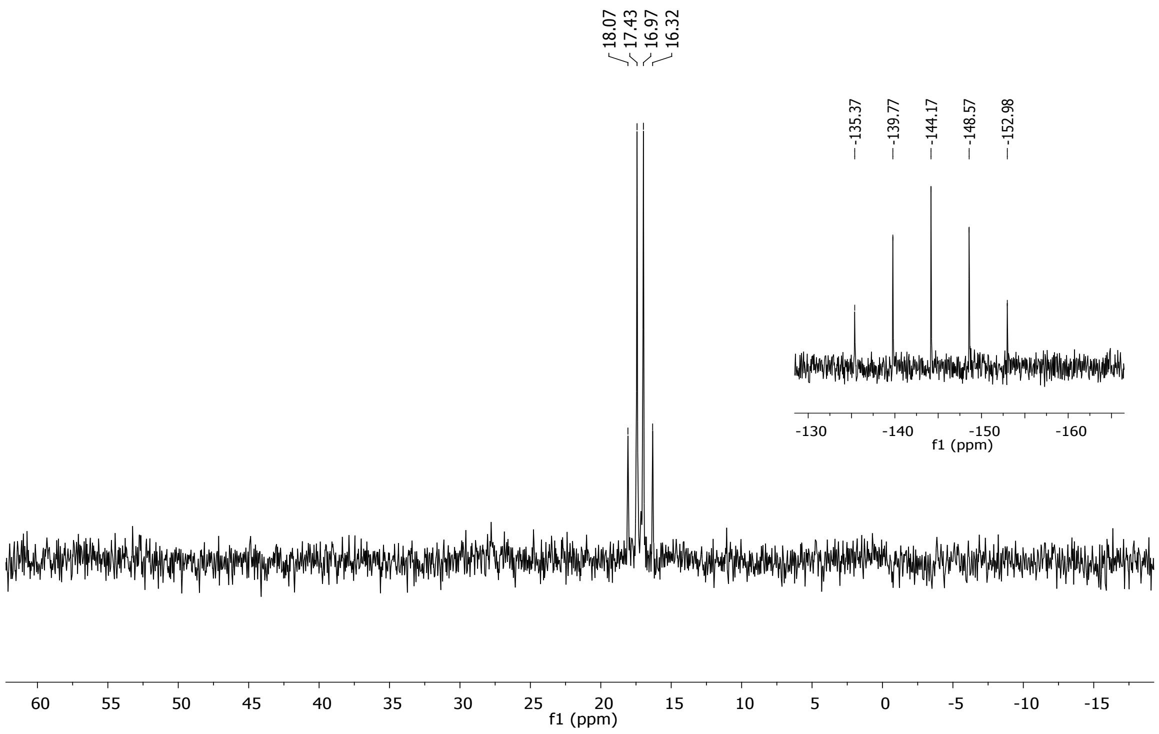

^{19}F NMR

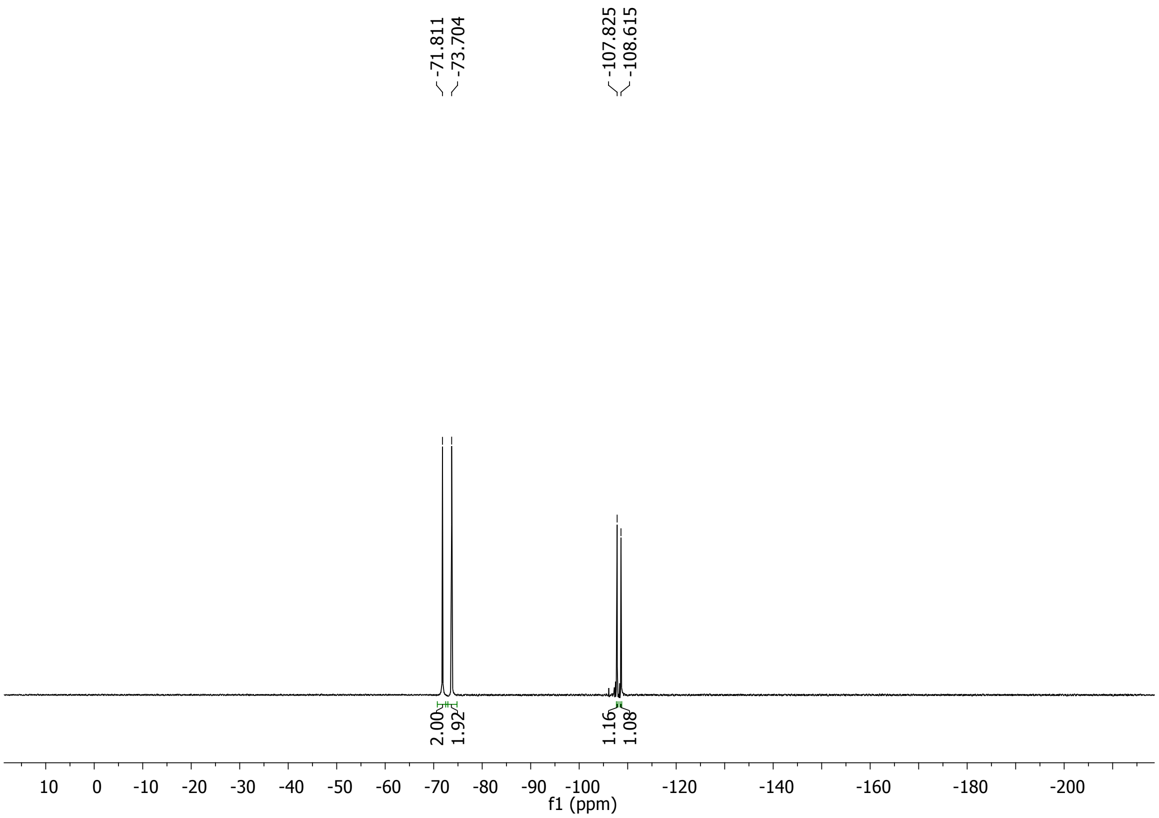

^{13}C NMR

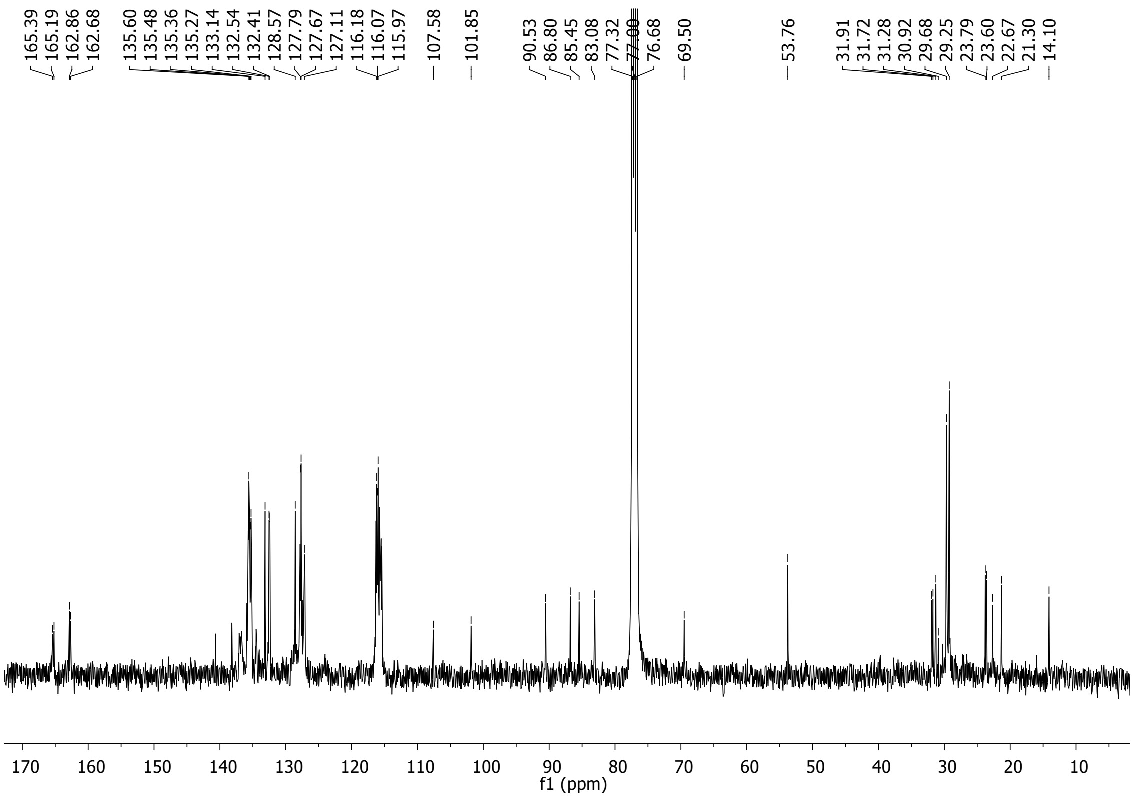

165.39
165.19
162.86
162.68
135.60
135.48
135.36
135.27
133.14
132.54
132.41
128.57
127.79
127.67
127.11
116.18
116.07
115.97
107.58
101.85
90.53
86.80
85.45
83.08
77.32
77.00
76.68
69.50
53.76
31.91
31.72
31.28
30.92
29.68
29.25
23.79
23.60
22.67
21.30
14.10
f1 (ppm)

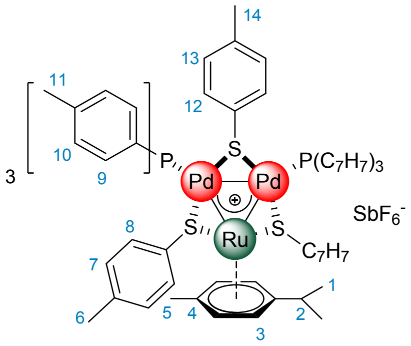
3
P
Pd
Pd
S
$P(C_7H_7)_3$
Ru
S
S
C_7H_7
SbF_6^-
1
2
3
4
5
6
7
8
9
10
11
12
13
14

^{1}H NMR

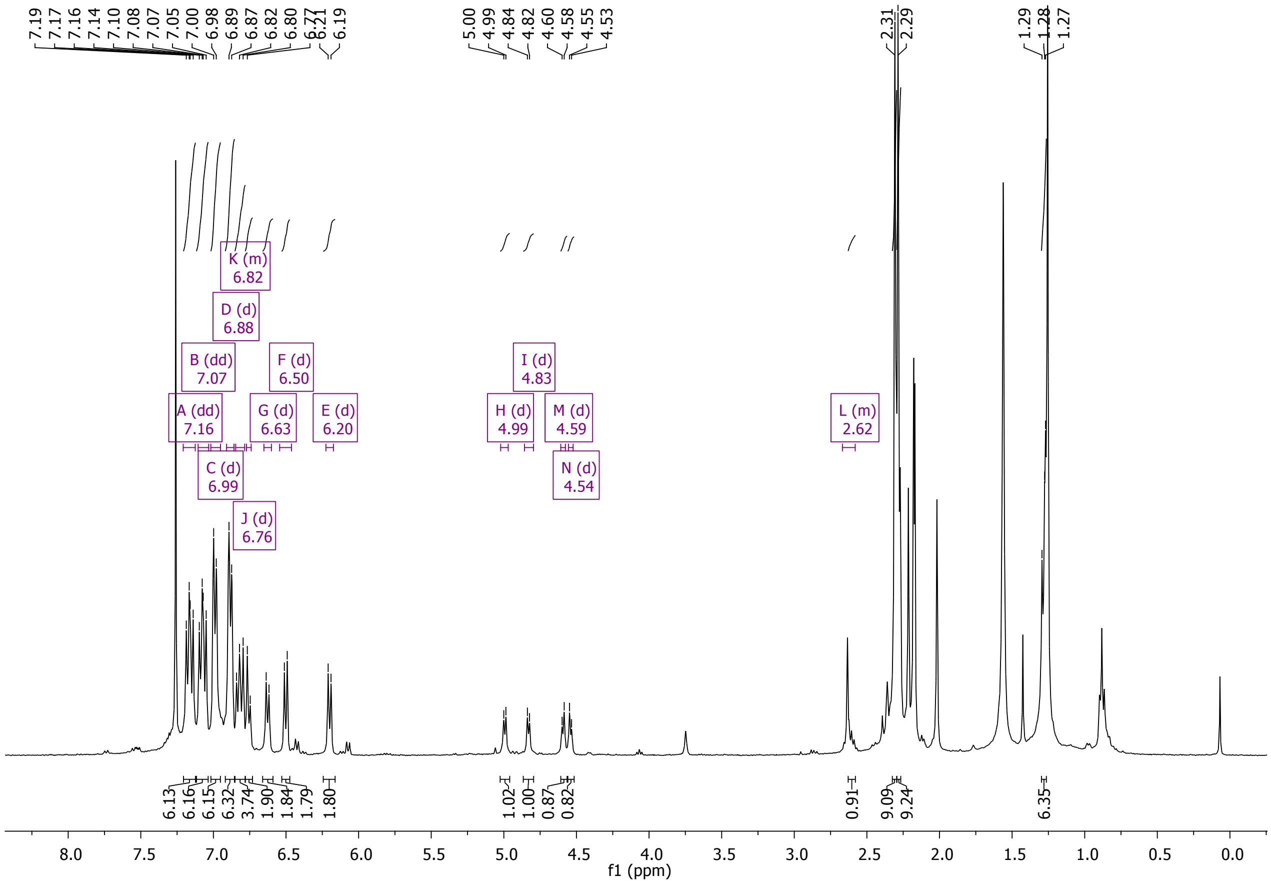
A (dd) 7.16
B (dd) 7.07
C (d) 6.99
D (d) 6.88
E (d) 6.20
F (d) 6.50
G (d) 6.63
H (d) 4.99
I (d) 4.83
J (d) 6.76
K (m) 6.82
L (m) 2.62
M (d) 4.59
N (d) 4.54
f1 (ppm)

^{31}P NMR

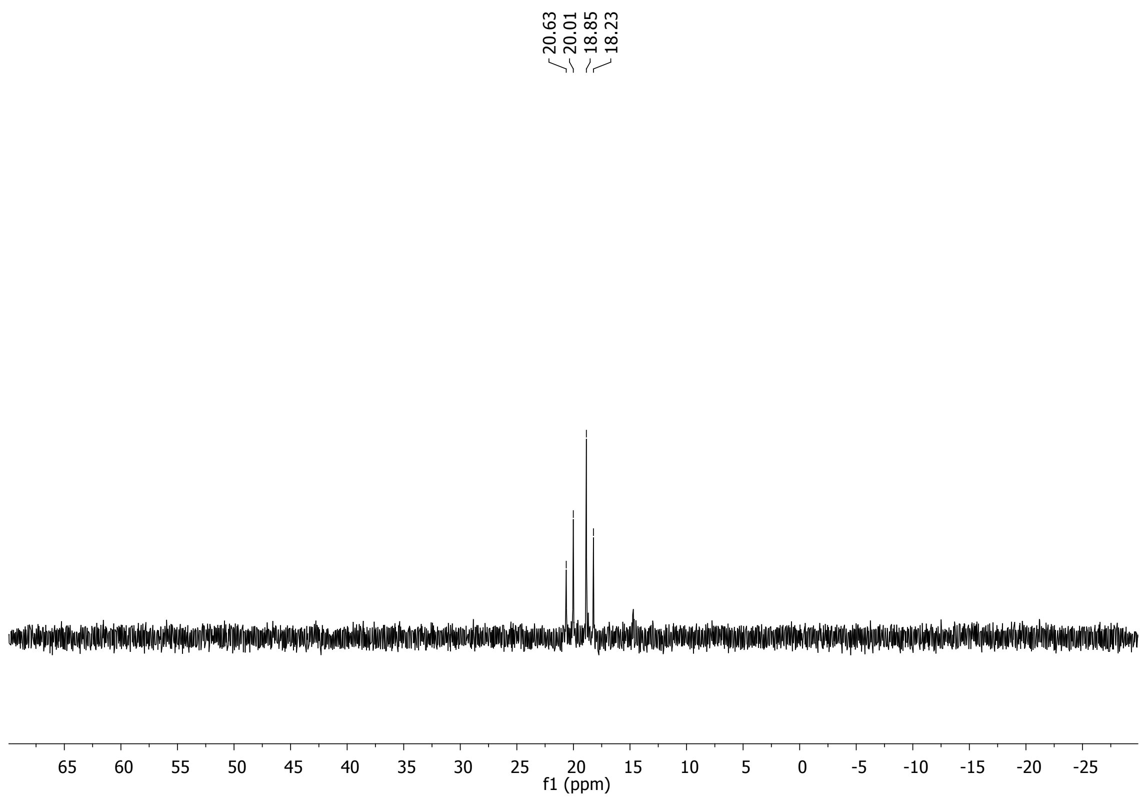

20.63
20.01
18.85
18.23
65
60
55
50
45
40
35
30
25
20
15
10
5
0
-5
-10
-15
-20
-25
f1 (ppm)

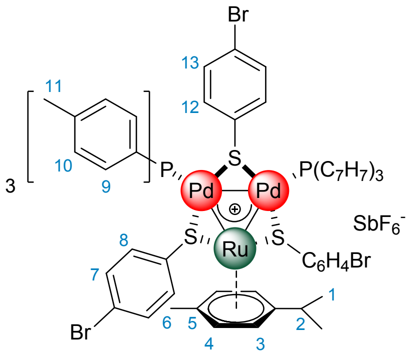

Br
13
12
11
10
9
3
P
Pd
Pd
S
P(C7H7)3
SbF6-
8
7
S
Ru
S
C6H4Br
Br
6
5
4
3
2
1

^{1}H NMR

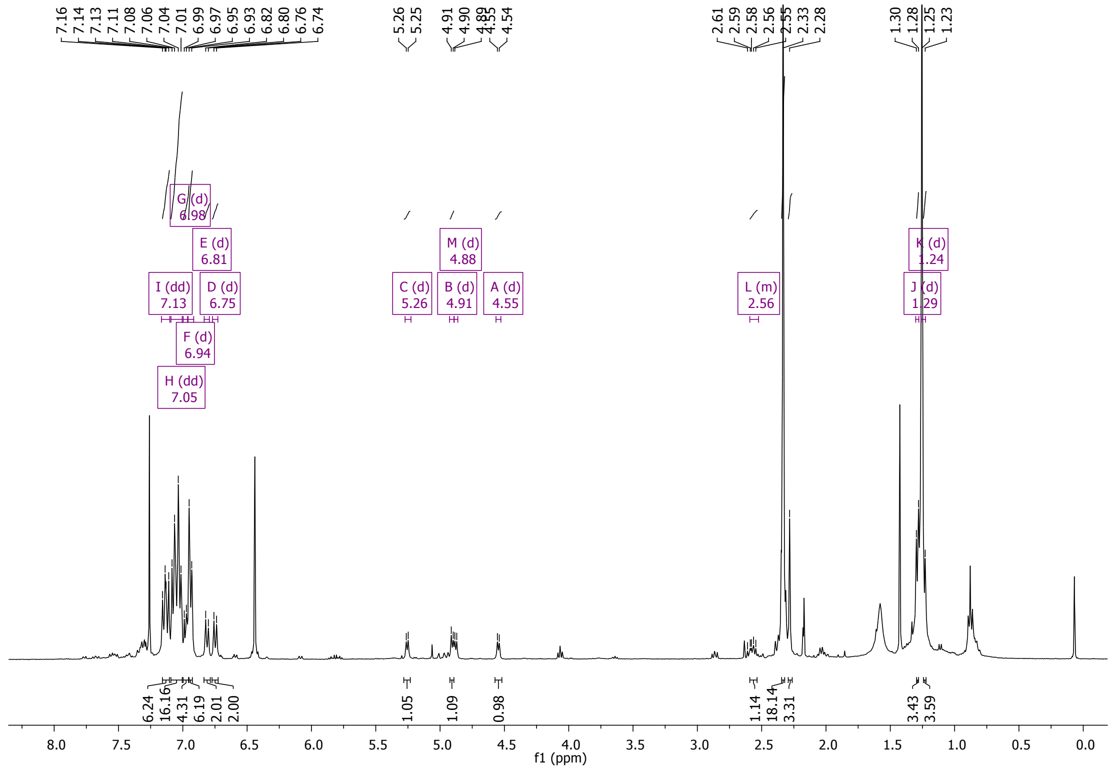

7.16
7.14
7.13
7.11
7.08
7.06
7.04
7.01
6.99
6.97
6.95
6.93
6.82
6.80
6.76
6.74
5.26
5.25
4.91
4.90
4.89
4.55
4.54
2.61
2.59
2.58
2.56
2.55
2.33
2.28
1.30
1.28
1.25
1.23
G (d) 6.98
E (d) 6.81
I (dd) 7.13
D (d) 6.75
F (d) 6.94
H (dd) 7.05
M (d) 4.88
C (d) 5.26
B (d) 4.91
A (d) 4.55
L (m) 2.56
K (d) 1.24
J (d) 1.29
6.24
16.16
4.31
6.19
2.01
2.00
1.05
1.09
0.98
1.14
18.14
3.31
3.43
3.59
f1 (ppm)

^{31}P NMR

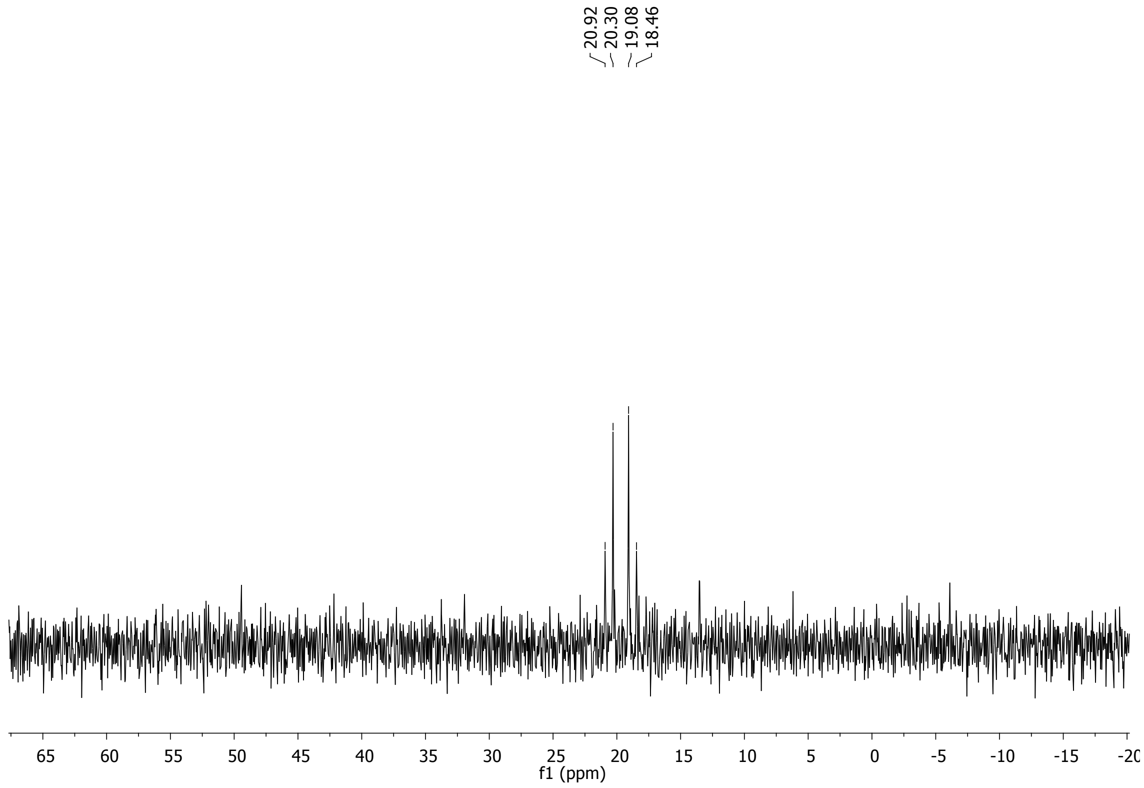
20.92
20.30
19.08
18.46
65
60
55
50
45
40
35
30
25
20
15
10
5
0
-5
-10
-15
-20
f1 (ppm)

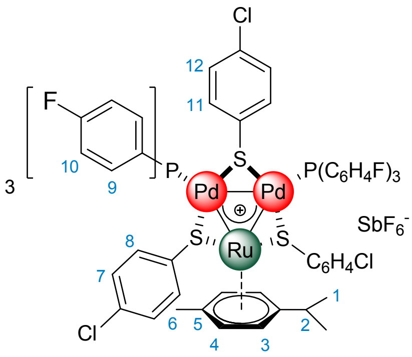

Cl
12
11
F
10
9
3
P
S
Pd
Pd
P(C6H4F)3
SbF6-
S
Ru
S
C6H4Cl
8
7
Cl
6
5
4
3
2
1

^{1}H NMR

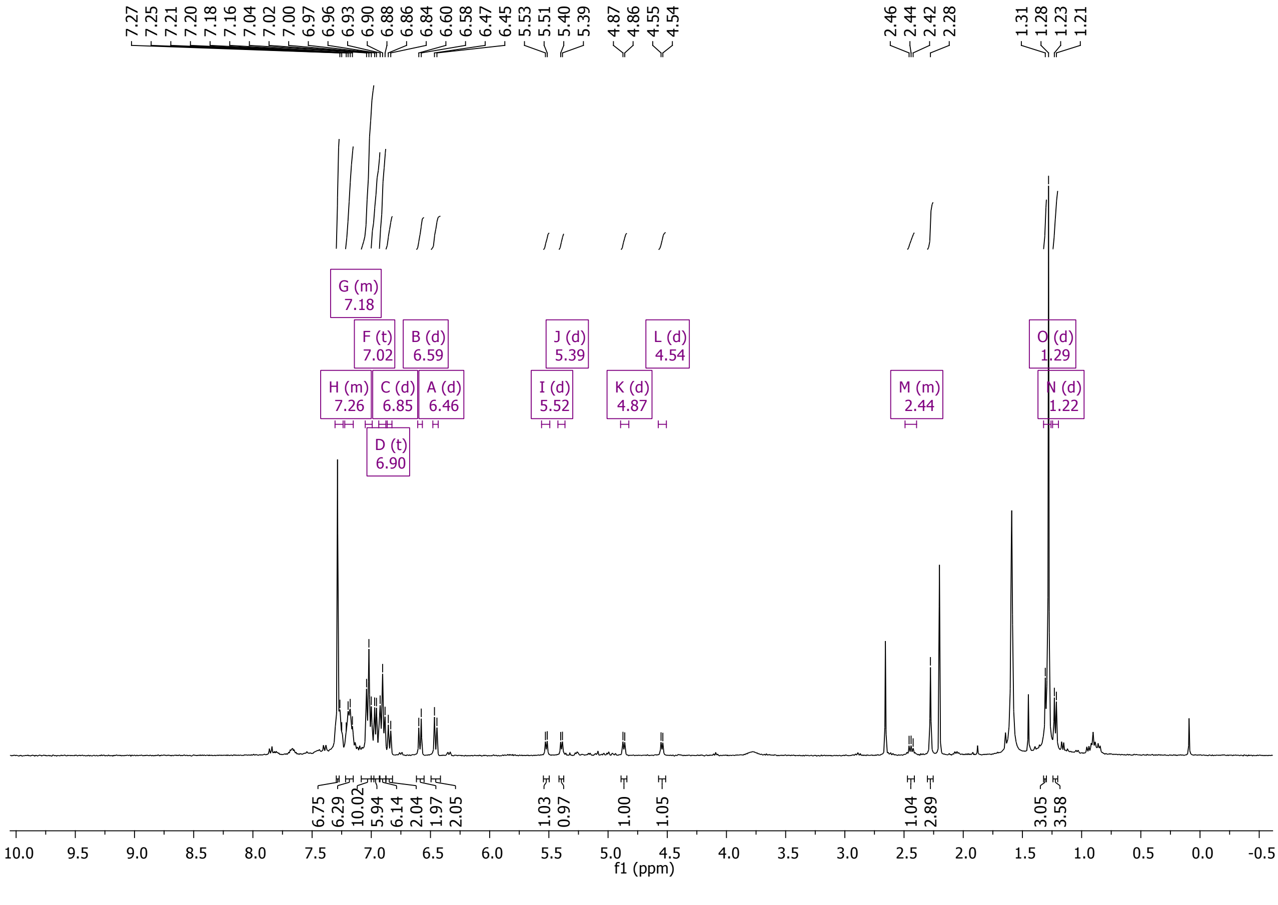

7.27 7.25 7.21 7.20 7.18 7.16 7.04 7.02 7.00 6.97 6.96 6.93 6.90 6.88 6.86 6.84 6.60 6.58 6.47 6.45 5.53 5.51 5.40 5.39 4.87 4.86 4.55 4.54 2.46 2.44 2.42 2.28 1.31 1.28 1.23 1.21
G (m) 7.18
F (t) 7.02
B (d) 6.59
J (d) 5.39
L (d) 4.54
O (d) 1.29
H (m) 7.26
C (d) 6.85
A (d) 6.46
I (d) 5.52
K (d) 4.87
M (m) 2.44
N (d) 1.22
D (t) 6.90
6.75 6.29 10.02 5.94 6.14 2.04 1.97 2.05 1.03 0.97 1.00 1.05 1.04 2.89 3.05 3.58
10.0 9.5 9.0 8.5 8.0 7.5 7.0 6.5 6.0 5.5 5.0 4.5 4.0 3.5 3.0 2.5 2.0 1.5 1.0 0.5 0.0 -0.5
f1 (ppm)

^{31}P NMR

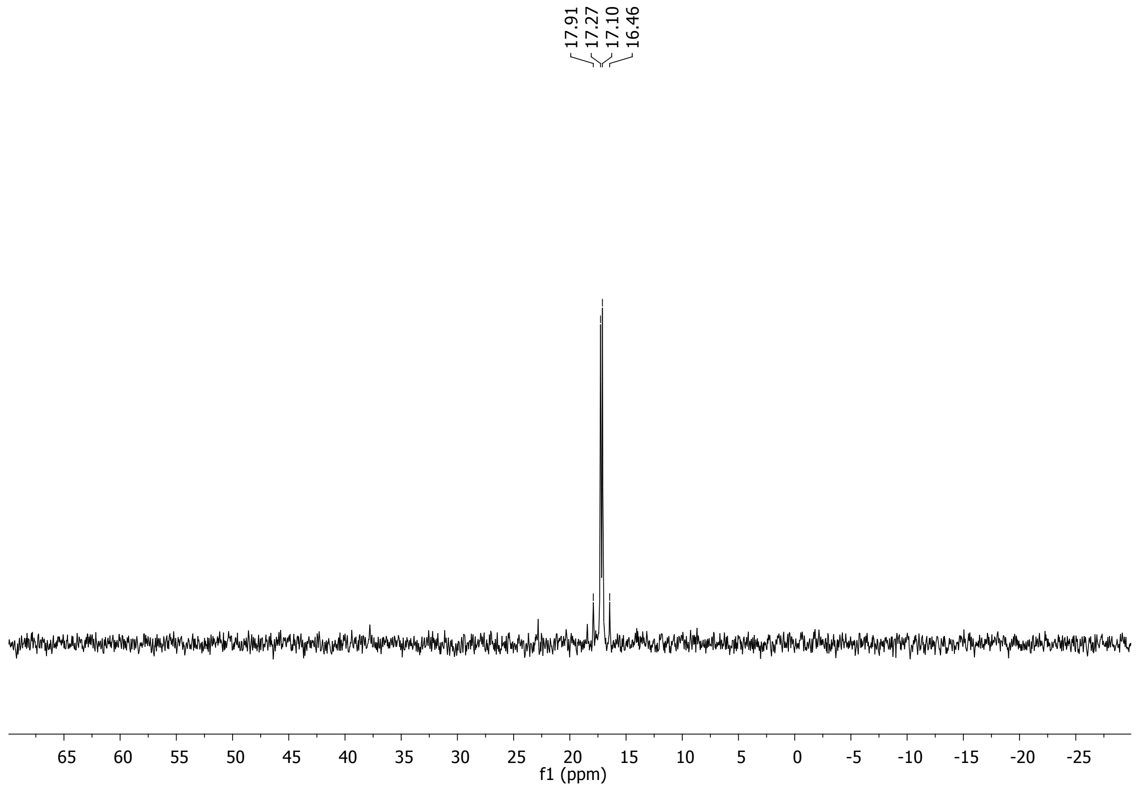

^{19}F NMR

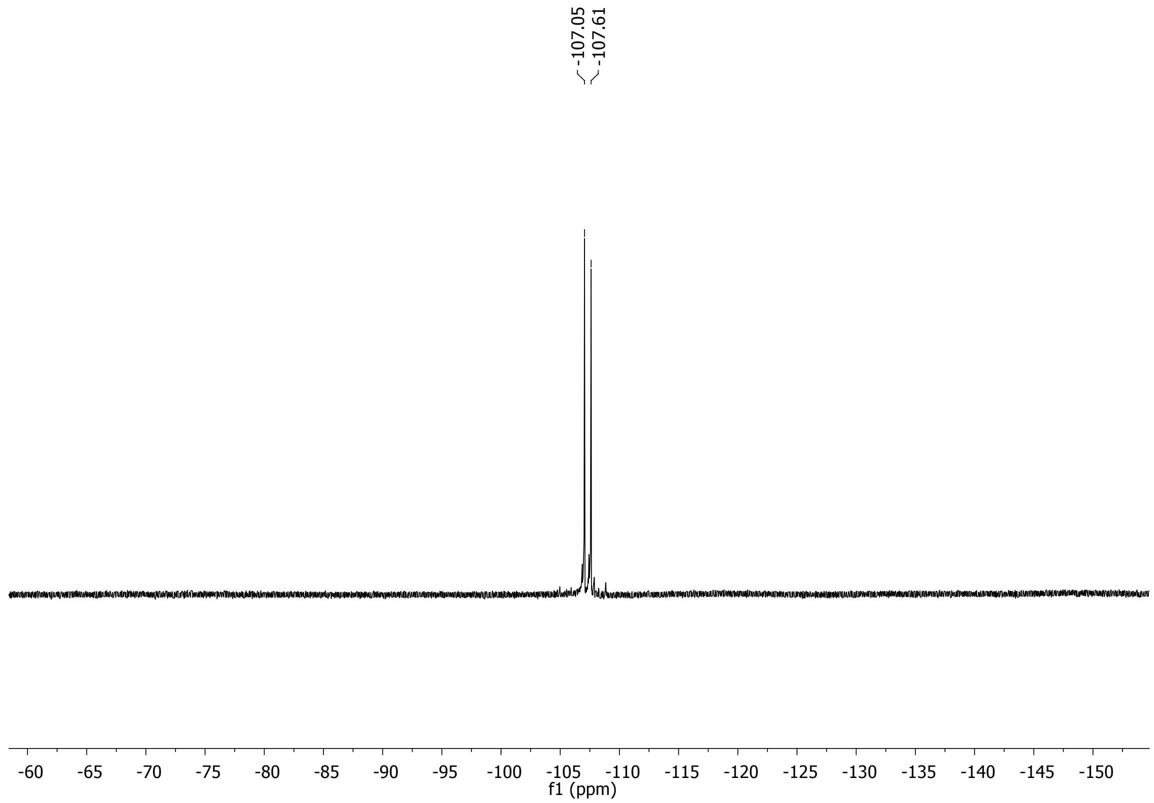

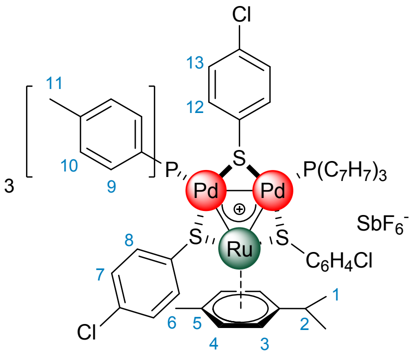

¹H NMR

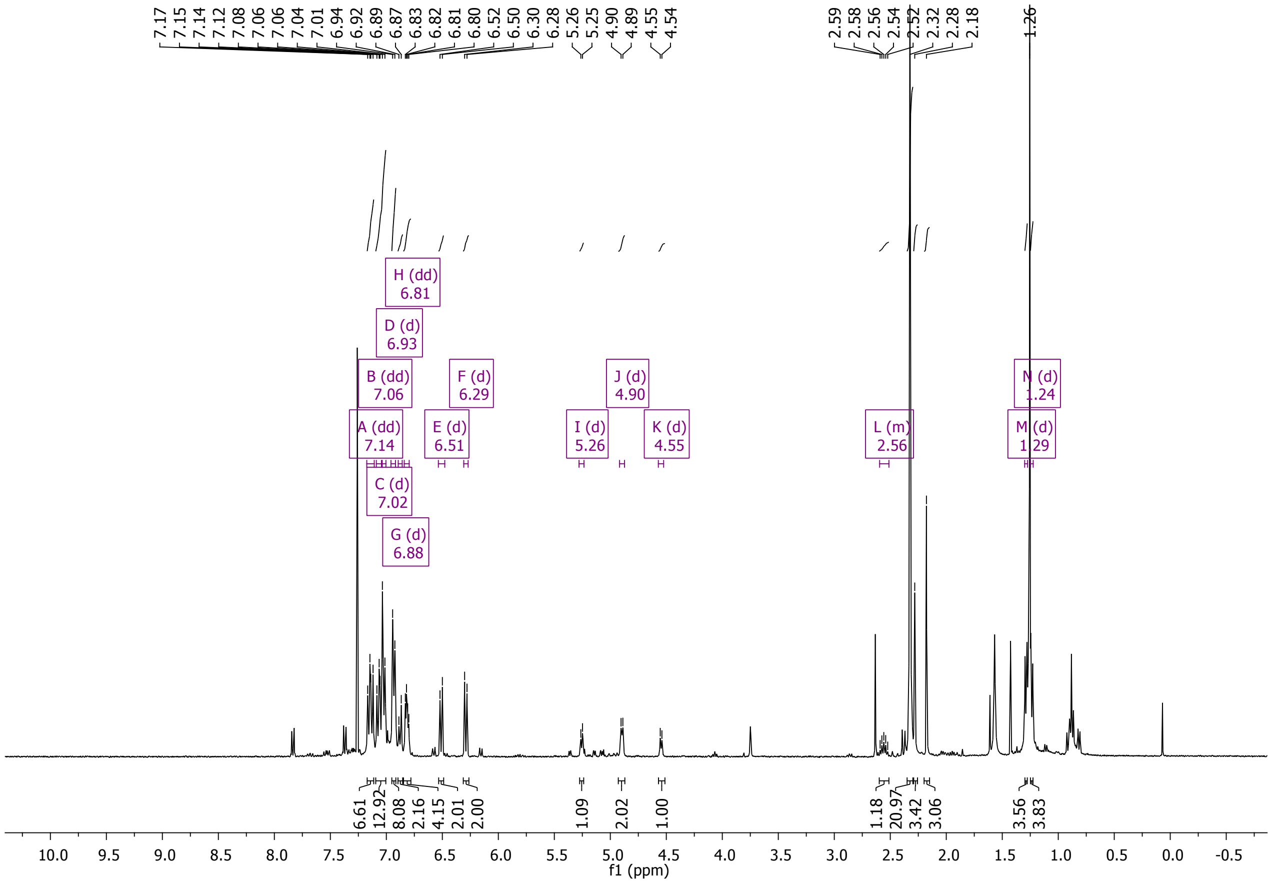

^{31}P NMR

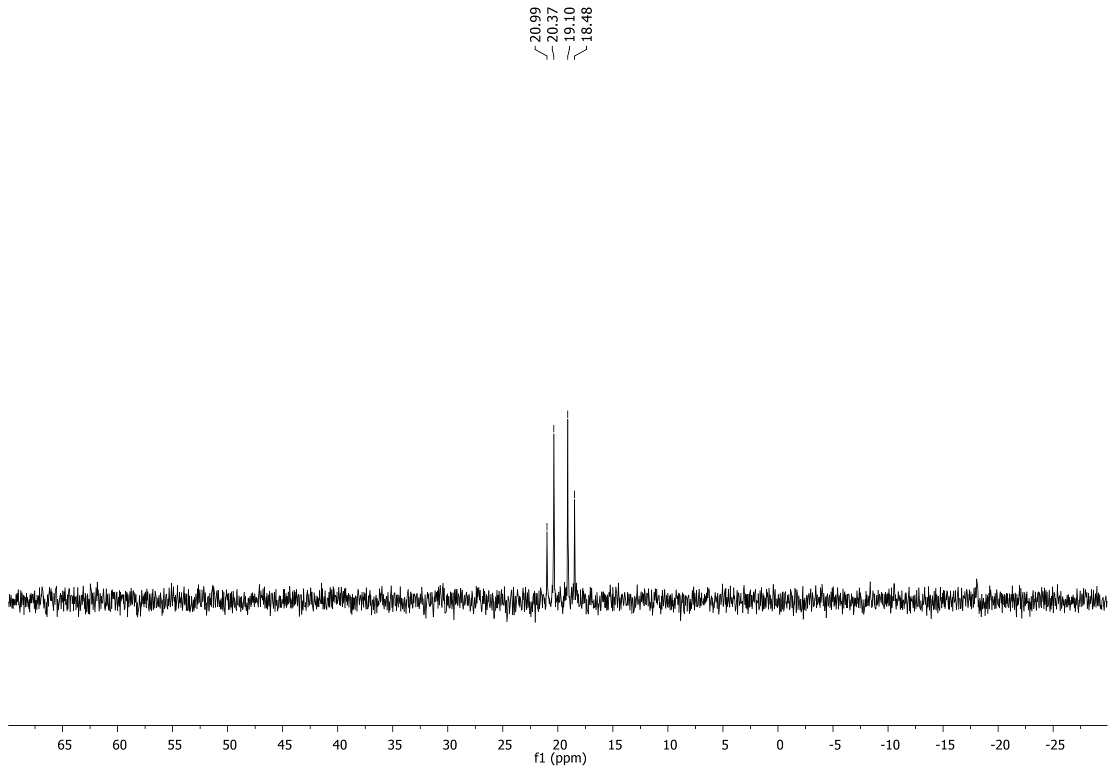

^{13}C NMR

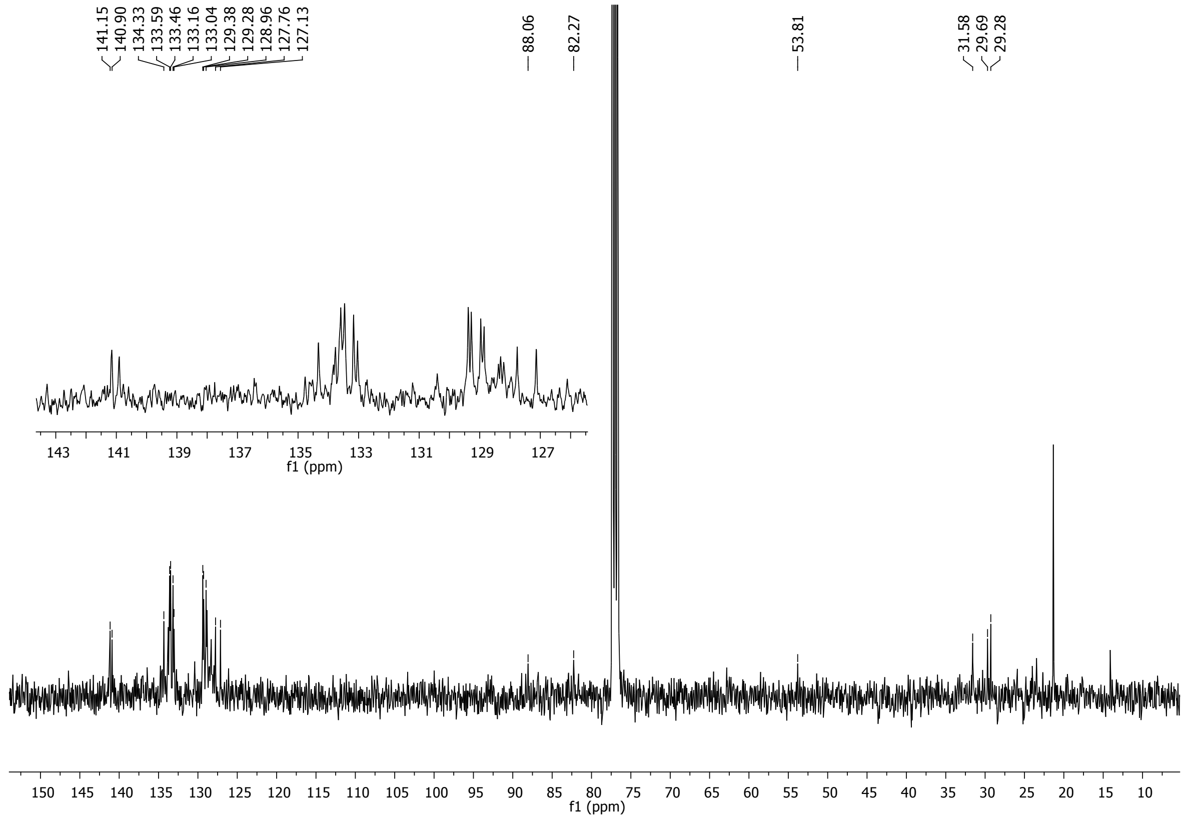

8. Cartesian coordinates of computed structures (PBE0-GD3BJ/6-31g+* (H,C,P,S)/Def2-TZVP (Ru,Pd)

Optimized structure of 1a compound

46	1.473572000	0.414551000	-0.231502000
46	-1.177237000	-0.232544000	-0.395324000
44	-0.391682000	2.465369000	-0.524558000
16	1.188316000	2.230414000	1.190161000
16	-2.096267000	1.520063000	0.756546000
16	0.561028000	-1.260404000	-1.515569000
15	3.700389000	-0.126315000	-0.105574000
15	-2.678230000	-1.959243000	-0.113580000
6	4.230384000	-1.469835000	-1.224236000
6	4.228935000	-0.631079000	1.562081000
6	0.599474000	1.519389000	2.714140000
6	-0.275020000	2.256197000	3.517114000
1	-0.654114000	3.211600000	3.165718000
6	3.821451000	-1.394688000	-2.561575000
1	3.176331000	-0.580568000	-2.882618000
6	3.763638000	-1.851315000	2.072779000
1	3.172839000	-2.514594000	1.447249000
6	4.228998000	-2.356009000	-3.479011000
1	3.909735000	-2.283057000	-4.515397000
6	-1.431071000	2.953193000	-2.403812000
1	-2.303936000	2.462915000	-2.819797000
6	-4.450981000	-1.630379000	-0.428295000
6	-4.085267000	1.500842000	-1.206151000
1	-3.452164000	0.802581000	-1.747337000
6	-2.938148000	-3.454382000	4.245719000

1	-3.012109000	-3.797552000	5.274294000
6	4.805993000	1.254780000	-0.570963000
6	-2.254672000	-3.381994000	-1.178238000
6	-1.610206000	3.992125000	-1.456101000
1	-2.622562000	4.243901000	-1.154134000
6	5.997411000	1.025750000	-1.269533000
1	6.274659000	0.016216000	-1.558794000
6	1.089938000	0.291343000	3.162462000
1	1.776513000	-0.276308000	2.543233000
6	0.721036000	-0.186533000	4.417318000
1	1.128535000	-1.133043000	4.764380000
6	-1.996028000	-1.953768000	2.604073000
1	-1.352508000	-1.110692000	2.362606000
6	5.055502000	-2.522194000	-0.820462000
1	5.397495000	-2.586679000	0.207936000
6	0.969064000	-2.781826000	-0.686857000
6	5.038619000	-3.415243000	-3.066318000
1	5.351316000	-4.172335000	-3.780488000
6	-5.101062000	-2.011791000	-1.604582000
1	-4.573261000	-2.578427000	-2.365936000
6	4.972856000	0.220079000	2.383868000
1	5.338809000	1.168274000	2.000346000
6	-0.138036000	2.466964000	-2.722526000
6	-0.505022000	4.622032000	-0.823266000
6	-3.623082000	-3.614164000	1.938206000
1	-4.249394000	-4.065393000	1.172301000
6	4.048954000	-2.215440000	3.384188000

1	3.692256000	-3.169072000	3.765577000
6	-2.091668000	-2.397450000	3.922225000
1	-1.510994000	-1.901167000	4.693377000
6	5.448719000	-3.495791000	-1.738393000
1	6.084135000	-4.314494000	-1.411539000
6	0.046604000	1.352160000	-3.702488000
1	0.969025000	0.800142000	-3.504345000
1	0.102998000	1.762065000	-4.718990000
1	-0.787256000	0.645741000	-3.662329000
6	-3.664621000	1.972518000	0.040645000
6	1.515600000	-3.824323000	-1.437281000
1	1.692224000	-3.688089000	-2.499616000
6	-2.748348000	-2.570996000	1.602007000
6	-0.653212000	1.763721000	4.762725000
1	-1.335755000	2.339397000	5.382308000
6	1.027812000	-4.173819000	1.288048000
1	0.821454000	-4.304624000	2.347780000
6	5.305397000	3.625290000	-0.533604000
1	5.036911000	4.636252000	-0.237295000
6	6.830780000	2.091635000	-1.600446000
1	7.752227000	1.903499000	-2.144780000
6	0.734905000	-2.958398000	0.679903000
1	0.322871000	-2.138013000	1.258424000
6	-0.656166000	5.738739000	0.181407000
1	0.242337000	5.713043000	0.813133000
6	4.464769000	2.563802000	-0.208178000
1	3.547659000	2.747485000	0.344507000

6	4.786854000	-1.360510000	4.203711000
1	5.004718000	-1.642147000	5.230328000
6	-3.709093000	-4.060048000	3.252227000
1	-4.386812000	-4.871337000	3.503852000
6	-2.101051000	-3.138607000	-2.550067000
1	-2.231583000	-2.130836000	-2.939151000
6	-4.495059000	2.823676000	0.774223000
1	-4.177746000	3.172483000	1.753702000
6	-0.151188000	0.545689000	5.220520000
1	-0.434711000	0.175329000	6.202307000
6	-5.723895000	3.220900000	0.248748000
1	-6.363346000	3.886572000	0.822560000
6	-5.163358000	-0.927509000	0.553651000
1	-4.667754000	-0.625241000	1.472995000
6	-6.504559000	-0.619754000	0.363456000
1	-7.045135000	-0.075297000	1.132637000
6	6.487536000	3.392036000	-1.234677000
1	7.140500000	4.220885000	-1.494218000
6	-5.313021000	1.895994000	-1.724920000
1	-5.639321000	1.509926000	-2.686794000
6	0.791715000	4.156951000	-1.195730000
1	1.657421000	4.555704000	-0.672094000
6	-6.446073000	-1.695087000	-1.794094000
1	-6.946449000	-2.007427000	-2.707079000
6	-2.005904000	-4.663764000	-0.682854000
1	-2.071407000	-4.862765000	0.381646000
6	5.246380000	-0.145506000	3.700965000

1	5.823694000	0.522847000	4.333917000
6	0.983683000	3.108939000	-2.122464000
1	1.986233000	2.735608000	-2.311181000
6	-7.149234000	-1.000071000	-0.813492000
1	-8.198365000	-0.759179000	-0.962372000
6	1.583253000	-5.213443000	0.540800000
1	1.818573000	-6.161998000	1.016115000
6	-1.639183000	-5.691063000	-1.550476000
1	-1.435642000	-6.680530000	-1.150290000
6	-1.750440000	-4.168644000	-3.415625000
1	-1.642137000	-3.967386000	-4.478161000
6	-6.132950000	2.762943000	-1.002139000
1	-7.093365000	3.069189000	-1.407405000
6	-0.688270000	7.082423000	-0.554709000
1	0.201524000	7.223186000	-1.178292000
1	-0.735407000	7.909812000	0.161686000
1	-1.569285000	7.147885000	-1.204553000
6	1.830992000	-5.032068000	-0.818541000
1	2.263254000	-5.837139000	-1.406775000
6	-1.871145000	5.573011000	1.088748000
1	-2.812159000	5.688230000	0.538292000
1	-1.859171000	6.338081000	1.872131000
1	-1.873854000	4.586385000	1.564210000
6	-1.521770000	-5.450799000	-2.916264000
1	-1.238268000	-6.255200000	-3.589639000

Optimized structure of a truncated model 1a compound

46	-0.085798000	1.577198000	-0.039280000
46	1.604512000	-0.554324000	0.062023000
44	-1.147678000	-1.001197000	-0.080211000
16	-1.726562000	0.757576000	1.353468000
16	0.353079000	-1.869160000	1.464861000
16	1.769977000	1.263528000	-1.367845000
15	-0.606990000	3.785979000	0.069680000
15	3.766216000	-1.243787000	0.320429000
6	-1.298089000	-2.774693000	-1.362649000
1	-0.709655000	-3.684487000	-1.297944000
6	-2.471135000	-2.654021000	-0.564068000
1	-2.756466000	-3.471902000	0.088879000
6	-0.820213000	-1.712413000	-2.159636000
6	-3.235342000	-1.460930000	-0.579333000
6	0.415925000	-1.854433000	-2.993703000
1	0.920183000	-0.893620000	-3.125213000
1	0.150699000	-2.241174000	-3.986126000
1	1.122960000	-2.553217000	-2.536445000
6	-4.490908000	-1.281678000	0.240082000
1	-4.558182000	-0.211142000	0.476272000
6	-2.771043000	-0.394125000	-1.406476000
1	-3.289738000	0.560425000	-1.369923000
6	-1.574776000	-0.497427000	-2.149493000
1	-1.206077000	0.363975000	-2.698856000
6	-5.703372000	-1.663083000	-0.616603000
1	-5.742208000	-1.084081000	-1.545939000
1	-6.630866000	-1.479659000	-0.063690000

1	-5.673794000	-2.726453000	-0.882618000
6	-4.475914000	-2.048840000	1.557930000
1	-4.534159000	-3.132507000	1.400937000
1	-5.345420000	-1.766922000	2.160263000
1	-3.571674000	-1.828716000	2.135568000
6	4.077600000	-2.884139000	1.072922000
1	5.153503000	-3.084398000	1.135378000
1	3.652620000	-2.912815000	2.080768000
1	3.607058000	-3.670009000	0.475084000
6	4.726638000	-1.331152000	-1.236552000
1	4.275004000	-2.070602000	-1.905053000
1	4.700687000	-0.362420000	-1.743527000
1	5.768716000	-1.612192000	-1.045535000
6	4.768244000	-0.140115000	1.384076000
1	4.327145000	-0.099131000	2.384627000
1	5.799691000	-0.502899000	1.462352000
1	4.777880000	0.872494000	0.972589000
6	0.525877000	4.999235000	-0.700105000
1	1.494733000	4.976209000	-0.192483000
1	0.679315000	4.743688000	-1.752994000
1	0.116746000	6.013975000	-0.633641000
6	0.641012000	-3.641455000	1.173813000
1	1.379432000	-3.996776000	1.897013000
1	-0.294634000	-4.182183000	1.343366000
1	1.001436000	-3.837172000	0.162205000
6	-0.839949000	4.446086000	1.760298000
1	-1.113281000	5.507234000	1.734369000

1	-1.636151000	3.885477000	2.259296000
1	0.081314000	4.329053000	2.338952000
6	-0.913580000	0.700184000	2.980354000
1	-1.353260000	-0.113168000	3.563812000
1	0.161661000	0.538026000	2.889308000
1	-1.103345000	1.649993000	3.487367000
6	-2.212209000	4.139374000	-0.736044000
1	-2.160414000	3.878767000	-1.797552000
1	-2.990812000	3.531173000	-0.265309000
1	-2.477815000	5.198735000	-0.642007000
6	3.106681000	2.392173000	-0.863985000
1	4.066515000	1.970544000	-1.177323000
1	3.110446000	2.557775000	0.214837000
1	2.972242000	3.345266000	-1.382315000

9. Two virtual σ-aromatic MOs

In this model, all ligands are replaced by hydrogen atoms and TM-coordinated benzene ring is removed. Otherwise, σ-aromatic character of these MOs is not visually readable due to the multicentre nature of MOs.

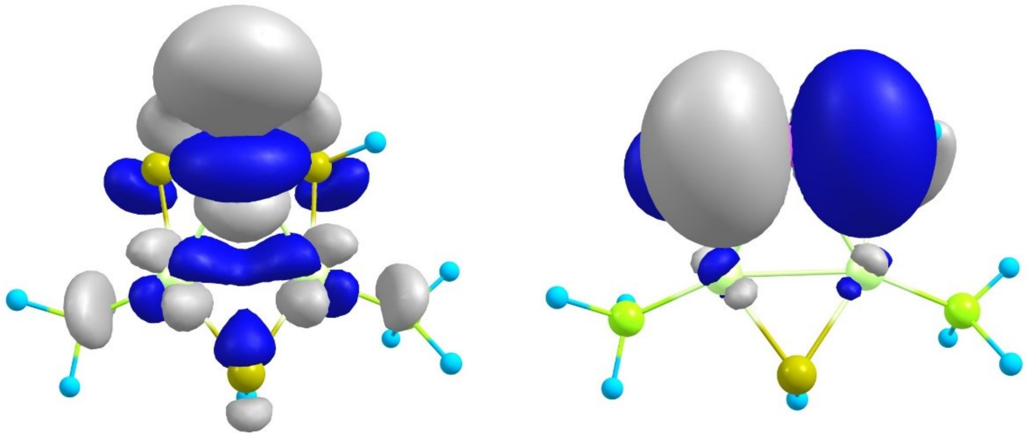

Virtual σ-aromatic MOs of the truncated model.

10. References

[1] Lausi A., Polentarutti M., Onesti S., Plaisier J. R., Busetto E., Bais G., Barba L., Cassetta A., Campi G., Lamba D., Pifferi A., Mande S. C., Sarma D. D., Sharma S. M., Paolucci G. (2015). Status of the crystallography beamlines at Elettra. The European Physical Journal Plus, 130(43), 1-8.

[2] Kabsch W. (2010). XDS. Acta Crystallographica Section D, 66(2), 125-132.

[3] Winn, M. D., Ballard, C. C., Cowtan, K. D., Dodson, E. J., Emsley, P., Evans, P. R., Keegan, R. M., Krissinel, E. B., Leslie, A. G. W., McCoy, A., McNicholas, S. J., Murshudov, G. N., Pannu, N. S., Potterton, E. A., Powell, H. R., Read, R. J., Vagin, A. & Wilson, K. S. (2011). Overview of the CCP4 suite and current developments. Acta Crystallographica Section D. 67, 235-242.

[4] Evans P. R. and Murshudov G. N. How good are my data and what is the resolution?. (2013). Acta Crystallographica Section D. 69, 1204-1214

[5] Sheldrick G. M. (2015). SHELXT – Integrated space-group and crystal-structure determination. Acta Crystallographica Section A, 71, 3-8.

[6] Sheldrick G. M. (2015). Crystal structure refinement with SHELXL. Acta Crystallographica Section C, 71, 3-8.

[7] Dolomanov, O.V., Bourhis, L.J., Gildea, R.J, Howard, J.A.K. & Puschmann, H. (2009), J. Appl. Cryst. 42, 339-341.
